# Evaluating performance of the 2019 EULAR/ACR, 2012 SLICC, and 1997 ACR criteria for classifying adult-onset and childhood-onset systemic lupus erythematosus: A systematic review and meta-analysis

**DOI:** 10.3389/fmed.2022.1093213

**Published:** 2022-12-22

**Authors:** Butsabong Lerkvaleekul, Pantira Chobchai, Sasivimol Rattanasiri, Soamarat Vilaiyuk

**Affiliations:** ^1^Rheumatology Division, Department of Pediatrics, Faculty of Medicine Ramathibodi Hospital, Mahidol University, Bangkok, Thailand; ^2^Section for Clinical Epidemiology and Biostatistics, Faculty of Medicine Ramathibodi Hospital, Mahidol University, Bangkok, Thailand

**Keywords:** connective tissue disease, classification, juvenile, diagnostic, American College of Rheumatology, Systemic Lupus International Collaborating Clinics, European League Against Rheumatism, systemic lupus erythematosus

## Abstract

**Introduction:**

The American College of Rheumatology (ACR) 1997, Systemic Lupus International Collaborating Clinics (SLICC) 2012, and European League Against Rheumatism (EULAR)/ACR 2019 SLE criteria are often used to classify patients with adult-onset and childhood-onset systemic lupus erythematosus (SLE) in clinical practice because there are currently no diagnostic criteria for SLE. However, there is scarce evidence regarding which criteria are best for diagnosing patients with adult-onset and childhood-onset SLE.

**Methods:**

We searched Medline and Scopus databases for English-language articles from inception through October 2021. Data were extracted from the included publications by two independent reviewers. We performed bivariate meta-analysis with a random-effects model to pool diagnostic parameters. Meta-regression and subgroup analyses were performed to explore heterogeneity sources. We used network meta-analysis to compare diagnosis performance among the three criteria and ranked them in descending order. Publication bias was assessed using Deeks’ funnel plot.

**Results:**

We included 29 studies for systematic review and meta-analysis. Of these, 18 studies involved adult-onset SLE and 11 studies involved childhood-onset SLE. The pooled sensitivities of the three criteria for diagnosis of adult-onset SLE were comparable between SLICC 2012 and EULAR/ACR 2019 [95.86, 95% confidence interval (CI) 92.28–97.81 vs. 94.79, 95% CI 92.03–96.63]; pooled specificity was highest in ACR 1997 (92.24, 95% CI 87.06–95.46). In childhood-onset SLE, pooled sensitivity was highest in SLICC 2012 (93.76, 95% CI 89.45–96.39), and pooled specificity was highest in ACR 1997 (95.89, 95% CI 91.73–98.00). In network meta-analysis, the pooled diagnostic odds ratio ranked highest for EULAR/ACR 2019 (131.570, 95% CI 61.50–281.47) in adult-onset SLE and ranked highest for SLICC 2012 (191.07, 95% CI 76.06–480.01) in childhood-onset SLE. Deeks’ funnel plot showed no publication bias.

**Conclusion:**

We found that the diagnostic performance of the ACR 1997, SLICC 2012, and EULAR/ACR 2019 criteria differed between adult-onset and childhood-onset SLE. EULAR/ACR 2019 performed best for adult-onset SLE and SLICC 2012 was best for childhood-onset SLE in classifying patients with SLE.

**Systematic review registration:**

[www.ClinicalTrials.gov], identifier [CRD420 21281586].

## 1 Introduction

Systemic lupus erythematosus (SLE) is a chronic multi-system connective tissue disease with diverse clinical phenotypes. Several factors are related to SLE pathogenesis, including innate and adaptive immune dysregulations, aberrant cytokines and autoantibodies, alteration of the microbiome, and vitamin D deficiency (1, 2). Interleukin (IL)-33 is one of the cytokines related to cellular damage, apoptosis, and immunological activation that play a significant role in the acute phase of SLE. It was found to be elevated in the serum of SLE patients relative to healthy controls (3, 4). A previous study on lupus-prone mice revealed that blocking IL-33 can expand regulatory T cells (Tregs) and reduce Th17 cells and proinflammatory cytokines, thereby protecting against SLE (5). The role of Th17 cells in SLE involves vascular inflammation, recruitment of neutrophils, stimulating B cells, and promoting autoantibody production (6, 7). There were reported in previous studies that active SLE patients had a higher level of Th17 cells, and the imbalance of Tregs and Th17 cells related to disease activity and severity of SLE (8–10). An imbalance in the gut microbial community may also contribute to the SLE disease activity, which was supported by a recent study that showed restoring the balance of gut microbiota, lower systemic inflammation, and improved SLE disease activity after synbiotic supplementation (11). Vitamin D deficiency was also associated with microbiome alteration and integrity of the gut barrier, which may cause autoimmune disease. In addition, vitamin D affects immune cells by suppressing Th17 and Th1 cell responses and enhancing the development of Tregs (2). Previous studies showed a correlation between low vitamin D levels and high disease activity in SLE patients, and vitamin D seems to be associated with the risk of acquiring the disease in genetically susceptible individuals (12). Regarding the complex disease mechanisms described above, SLE patients exhibit unique clinical characteristics and therapy responses.

Diagnosis and treatment of SLE are difficult owing to various disease presentations, changing clinical characteristics, and unpredictable disease progression (13). Until now, three important classification criteria have been used for classifying SLE: the American College of Rheumatology (ACR) 1997, the Systemic Lupus International Collaborating Clinics (SLICC) 2012, and the European League Against Rheumatism (EULAR)/ACR 2019 SLE criteria. Although these SLE classification criteria were developed primarily for research purposes, they are also commonly used in clinical practice because there are currently no diagnostic criteria for SLE (14).

The ACR 1997 criteria have been widely used in clinical practice for nearly 30 years. The ACR 1997 requires 4 of 11 items to be met, with equal weight given to clinical and immunologic items (15). Clinicians and researchers have considerable concerns about these criteria, especially the limited sensitivity, absence of numerous cutaneous and neurological symptoms, omission of low complement levels, and the inclusion of only typical features of SLE. Major changes proposed in the 2012 SLICC criteria were the expansion of cutaneous and neurological criteria, allocation of cytopenia and autoantibodies in individual criteria, inclusion of alopecia and hypocomplementemia, and patients with only lupus nephritis with antinuclear antibody (ANA) or anti-dsDNA (16). To diagnose SLE using the SLICC 2012 criteria, at least four criteria must be met, with at least one clinical item and one immunological item. The benefits of SLICC 2012 over ACR 1997 have been widely reported, including better sensitivity, improved diagnostic accuracy, and eligibility for early diagnosis of patients with both adult-onset and childhood-onset SLE (17–22). However, the limitation of SLICC 2012 is mainly owing to its low specificity (16, 22, 23). The EULAR/ACR 2019 criteria were subsequently developed, to improve specificity and maintain sensitivity. The EULAR/ACR 2019 criteria involves a scoring system that requires ANA positivity with an immunofluorescence titer of 1:80 or greater as an entry criterion. The patient is classified as having SLE if the total score equals or exceeds 10 in weighted scores of clinical and laboratory domains (24). In adult-onset SLE, these new criteria outperformed previous criteria regarding sensitivity and specificity in a validation cohort (25). However, recent studies have found that the sensitivity of EULAR/ACR 2019 is similar to or lower than that of SLICC 2012 whereas the specificity of EULAR/ACR 2019 is similar to or lower than that of ACR 1997 in adult-onset SLE (17, 26). Conflicting results have also been found in previous childhood-onset SLE studies. Some authors have found that EULAR/ACR 2019 is not the best tool for childhood-onset SLE diagnosis compared with SLICC 2012 and ACR 1997 because it has lower sensitivity than SLICC 2012 and lower specificity than ACR 1997 (27, 28). However, some studies have found that EULAR/ACR 2019 performs better and improves over time compared with SLICC 2012 and ACR 1997 (29, 30).

SLE is a heterogeneous disease with varying clinical symptoms, disease course, and disease onset and progression, which contribute to the different performance of these three criteria according to published scientific reports. There are currently limited data regarding which classification criteria are suitable for diagnosing patients with adult-onset and childhood-onset SLE in clinical practice worldwide. Therefore, we conducted a systematic review and meta-analysis to address this knowledge gap. In this study, we aimed to assess (1) performance of the ACR 1997, SLICC 2012, and EULAR/ACR 2019 criteria for classifying patients with adult-onset and childhood-onset SLE, (2) comparisons of the performance of three criteria for diagnosis of patients with adult-onset and childhood-onset SLE, and (3) differences in performance among these three criteria for adult-onset and childhood-onset SLE in clinical practice.

## 2 Materials and methods

This study was conducted and reported according to the Preferred Reporting Items for Systematic Review and Meta-analysis of Diagnostic Test Accuracy Studies (PRISMA-DTA) (31). This study was registered in PROPERO with ID number CRD42021281586.

### 2.1 Search strategy and study selection

We performed a literature search using Medline and Scopus databases from inception to October 2021. Search terms and search strategies were constructed based on population (P), Intervention (I), Comparator (C), and Outcome (O). These included combinations of the following keywords: (1) “SLE” or “systemic lupus erythematosus” or “lupus” and (2) “ACR” or “American College of Rheumatology” or “SLICC” or “Systemic Lupus International Collaborating Clinics” or “EULAR” or “European League Against Rheumatism” and (3) “criteria” or “classification.” Two independent reviewers (PC and BL) identified the relevant articles. After screening selected articles based on their title and abstract, we evaluated the full text of eligible articles to determine their relevance. We classified these as included, excluded, or requiring further evaluation. We contacted the primary investigator by email if a paper required further assessment. Disagreements were resolved in discussion or with the third reviewer (SV). Only full articles published in English language were included in the meta-analysis. The following inclusion criteria were used to determine eligibility of a study in this systematic review: (1) published articles assessing the performance of the 2019 EULAR/ACR, 2012 SLICC, or 1997 ACR criteria for classifying adult-onset or childhood-onset SLE; (2) studies that used the clinical diagnosis as the reference standard; and (3) availability of the number of true positives (TPs), false positives (FPs), true negatives (TNs), and false negatives (FNs) or available data that could be back-calculated from the sensitivity and specificity values. The exclusion criteria were: (1) review articles, case reports, letters to the editor, research protocols, and abstracts; and (2) studies with populations that overlapped with included studies.

### 2.2 Data extraction

We extracted the following study characteristics: the first author, year of publication, number of centers, setting, country, study design, ethnicity, index test (set of classification criteria), reference test, number of patients, and controls fulfilling ACR 1997, SLICC 2012, and EULAR/ACR 2019 criteria, clinical manifestations (neurological involvement, hematological involvement, skin involvement, renal involvement), immunological data, characteristic of patients, and controls (inclusion criteria, number, diagnosis, percentage of female patients, age of onset, age of diagnosis, duration of disease,% ANA positivity). Additionally, the number of TPs, FPs, TNs, and FNs were retrieved for data pooling. Two reviewers (PC and BL) independently extracted the data, and disagreements were resolved in discussion and with a third reviewer (SV) if necessary.

### 2.3 Risk of bias assessment

We used the Quality Assessment of Diagnostic Accuracy Studies-2 (QUADAS-2) (32) tool to assess the risk of bias and applicability for all included studies, conducted by two independent reviewers (PC and BL) (Supplementary Table 1). Conflicts were resolved in discussion.

### 2.4 Data analysis

We performed a bivariate meta-analysis with a random-effects model for pooling diagnostic parameters [i.e., sensitivity, specificity, likelihood ratio positive/negative, and diagnostic odds ratio (DOR)] using metandi, meta, midas commands in Stata (33). We used hierarchical summary receiver operating characteristic (ROC) curve analysis to construct summary receiver operating characteristic (SROC) curves (34). Between-study heterogeneity in sensitivity and specificity was assessed using Cochrane’s *Q*-test and Higgin’s *I*^2^ statistic. Heterogeneity was considered with a *Q*-test < 0.1 or *I*^2^ > 25%. We explored the sources of heterogeneity by fitting the covariates (i.e., ethnicity, disease phenotypes, % ANA positivity in controls, and time of diagnosis) one by one in DOR, incorporating both sensitivity and specificity in meta-regression and subgroup analyses according to that covariate. We performed a network meta-analysis to estimate and rank the probability of being the best diagnostic performance using a rankogram and the surface under the cumulative ranking curves (SUCRA) method. Analyses and network commands were conducted using Stata version 17.0 (StataCorp, College Station, TX, USA). Publication bias was assessed using Deeks’ funnel plot. *P* < 0.05 was considered statistically significant.

## 3 Results

### 3.1 Study selection and characteristics of included studies

**FIGURE 1:**
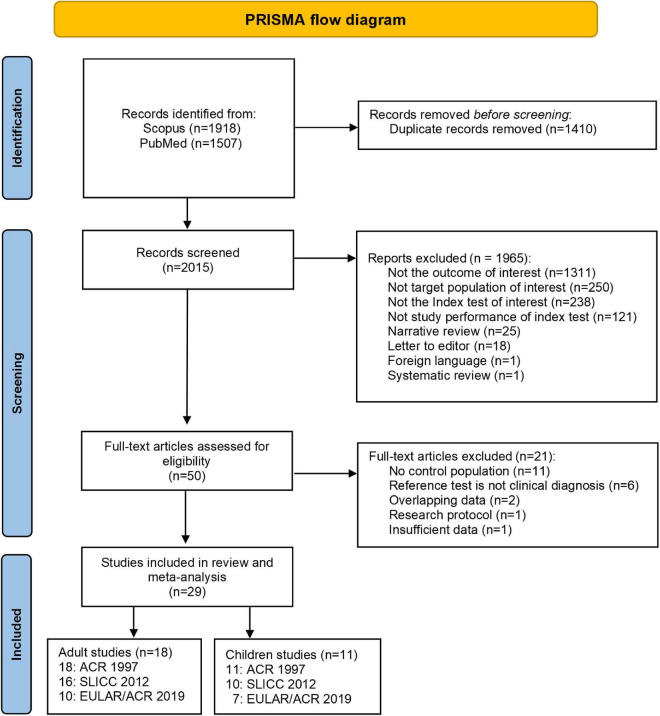
A PRISMA flow diagram summarizing the study selection process.

A total of 3,425 studies were identified in PubMed and Scopus. After deleting duplicates, 29 studies met the inclusion criteria (Figure 1). Of these, 18 studies (17, 18, 25, 26, 35–48) included patients with adult-onset SLE, and 11 studies (21, 22, 27, 29, 30, 49–54) included patients with childhood-onset SLE. For adult-onset SLE, 10 studies (17, 25, 26, 38, 41, 42, 45–48) assessed the three criteria (ACR 1997, SLICC 2012, and EULAR/ACR 2019), six studies (18, 36, 37, 39, 43, 44) assessed two criteria (ACR 1997 and SLICC 2012), and two studies (35, 40) assessed only one set of criteria (ACR 1997). Six childhood-onset SLE studies (27, 29, 30, 52–54) evaluated all three criteria (ACR 1997, SLICC 2012, and EULAR/ACR 2019), and five studies (21, 22, 49–51) evaluated two criteria (four studies evaluated ACR 1997 and SLICC 2012, and one study evaluated ACR 1997 and EULAR/ACR 2019). Only two included studies had more than one reference standard (one adult-onset and one childhood-onset SLE study). Dahlstrom et al. used fulfillment of the Fries diagnostic principle and/or ACR 82, and Smith et al. used ACR 1997, combined with clinical diagnosis as the reference standard (17, 52). One study was excluded because of insufficient data after three unsuccessful attempts to reach the author (55). Because diagnosing SLE as early as possible is important, as in actual practice, we selected the earliest time point with a confirmed diagnosis of SLE in studies with multiple data points to calculate the summary data.

Most of the 18 adult-onset SLE studies were conducted in an academic setting and used a retrospective design (Supplementary Table 2). Of these 18 studies, eight (17, 18, 26, 38, 41–44) were conducted in European (EU) countries, four (36, 45, 46, 48) were conducted in Asia, three studies (35, 40, 47) were in the United States (US), two studies (25, 37) were multinational, and one study (39) was conducted in Mexico. The participants were mostly female patients, with a mean age ranging from 30 to 50 years. The control group in each study had a different percentage and type of underlying autoimmune diseases. There were 11 childhood-onset SLE studies, mostly in tertiary and academic settings, with a retrospective study design; only one study had a cross-sectional design (50) (Supplementary Table 3). Most included studies were conducted in Brazil, the US, and the United Kingdom. The patients and controls were female individuals, with an average age between 10–15 and 7–11 years, respectively. The control group had various percentages and types of underlying autoimmune disease. The details of SLE characteristics differed between adult-onset and childhood-onset SLE studies.

### 3.2 Risk of bias and applicability

**FIGURE 2:**
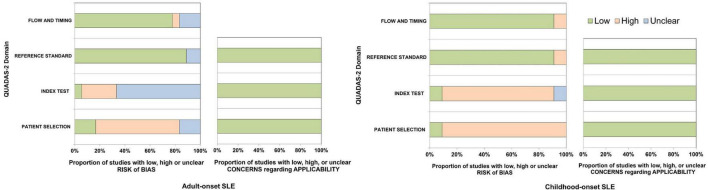
The quality assessment of diagnostic accuracy studies-2 for adult-onset and childhood-onset SLE studies.

The results of risk of bias and applicability in each study are presented in Figure 2. The included studies were not prospective cohorts, and most studies did not mention blinding of clinical diagnosis when applying the criteria. Additionally, we included two studies with more than one reference standard that contributed to the heterogeneity of reference test performance; these could result in a risk of bias. For adult-onset SLE studies, 12 (67%) had a high risk of bias as they used a case-control design, three studies (17%) had unclear risk of bias, and three studies (17%) had a low risk of bias in the patient selection domain. Most studies [12 (67%)] had an unclear risk of index tests because they had unclear reports of whether the results were interpreted without knowledge of the clinical diagnosis. Among the total, 16 (87%) and 14 (78%) studies had low risk in the reference standard and flow and timing domain. For childhood-onset SLE studies, the patient selection domain and index test domain were judged to have a high risk of bias consistent with adult studies [10 (91%) and 9 (82%)]. Most studies [10 (91%)] had a low risk of bias in both the reference standard and the flow and timing domains.

### 3.3 Diagnostic performance of the ACR 1997, SLICC 2012, and EULAR/ACR 2019 criteria

**FIGURE 3:**
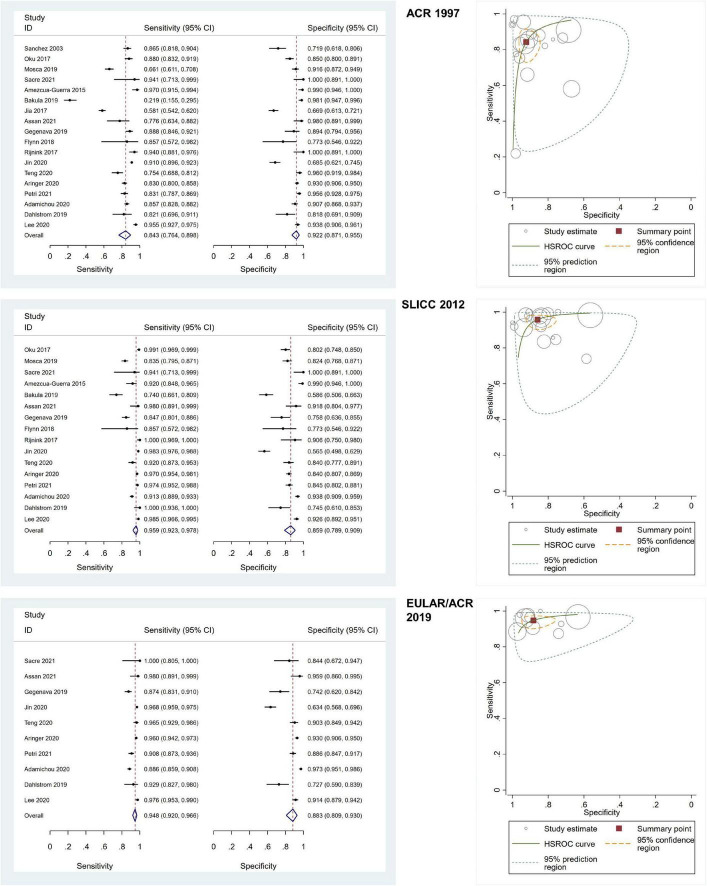
The forest plots depict the pooled sensitivity and specificity for the included adult-onset SLE studies and hierarchical summary receiver operating curve of the sensitivity and specificity for the American College of Rheumatology (ACR) 1997, Systemic Lupus International Collaborating Clinics (SLICC) 2012, and European Alliance of Associations for Rheumatology (EULAR)/ACR 2019.

**FIGURE 4:**
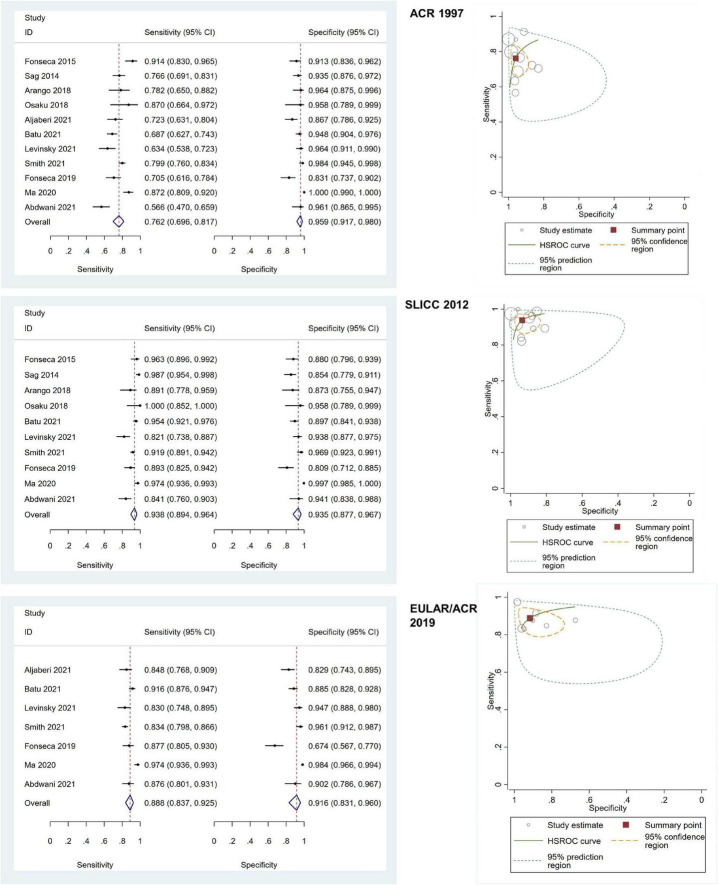
The forest plots depict the pooled sensitivity and specificity for the included childhood-onset SLE studies and hierarchical summary receiver operating curve of the sensitivity and specificity for the American College of Rheumatology (ACR) 1997, Systemic Lupus International Collaborating Clinics (SLICC) 2012, and European Alliance of Associations for Rheumatology (EULAR)/ACR 2019.

Of all included adult-onset SLE studies, the meta-analysis for ACR 1997, SLICC 2012, and EULAR/ACR 2019 comprised 6,445 patients, 5,550 patients, and 4,550 patients, respectively (Table 1). The pooled sensitivities and specificities of the ACR 1997, SLICC 2012, and EULAR/ACR 2019 criteria for the diagnosis of SLE were as follows: ACR 1997, 84.25 (95% CI 76.39–89.85) and 92.24 (95% CI 87.06–95.46); SLICC 2012, 95.86 (95% CI 92.28–97.81) and 85.95 (95% CI 78.88–90.92); EULAR/ACR 2019, 94.79 (95% CI 92.03–96.63) and 88.25 (95% CI 80.88–93.03), respectively. The area under the ROC curve (AUC) of ACR 1997 was 0.95 (95% CI 0.92–0.96) and similar between SLICC 2012 and EULAR/ACR 2019, which was 0.97 (95% CI 0.95–0.98). The SROC and forest plots of all adult-onset SLE studies are shown in Figure 3. For childhood-onset SLE studies, the pooled sensitivities and specificities of the ACR 1997, SLICC 2012, and EULAR/ACR 2019 criteria for diagnosis of SLE were 76.18 (95% CI 69.61–81.70) and 95.89 (95% CI 91.73–98.00) for ACR 1997, 93.76 (95% CI 89.45–96.39) and 93.49 (95% CI 87.68–96.67) for SLICC 2012 and 88.84 (95% CI 83.73–92.48) and 91.62 (95% CI 83.11–96.04) for EULAR/ACR 2019, respectively (Table 1). The AUC of SLICC 2012 was 0.98 (95% CI 0.96–0.99), followed by EULAR/ACR 2019 0.95 (95% CI 0.93–0.96), and ACR 1997 0.91 (95% CI 0.88–0.93). The summary ROC and forest plots of all childhood-onset SLE studies are shown in Figure 4. Heterogeneity was observed among all studies included in the meta-analysis in both adult-onset and childhood-onset SLE studies (Table 1).

**TABLE 1:** Diagnostic performance of the ACR 1997, SLICC 2012, and EULAR/ACR 2019 criteria.

Index test	Number of studies	Cases/ Participants	Pooled sensitivity (%) (95% CI)	Pooled specificity (%) (95% CI)	Pooled positive likelihood ratio (95% CI)	Pooled negative likelihood ratio (95% CI)	Pooled diagnostic odd ratio (95% CI)	The area under the curve (95% CI)	Heterogeneity: *I*^2^		*P*-value of Deeks
									**Sensitivity (%)**	**Specificity (%)**	
**Adult-onset systemic lupus erythematosus studies**											
ACR’97	(18)	6445/9807	84.25 (76.39–89.85)	92.24 (87.06–95.46)	10.86 (6.36–18.56)	0.17 (0.11–0.26)	63.64 (29.21-138.66)	0.95 (0.92–0.96)	88.29	80.63	0.32
SLICC’12	(16)	5550/8608	95.86 (92.28–97.81)	85.95 (78.88–90.92)	6.82 (4.45–10.46)	0.05 (0.03–0.09)	141.46 (59.74-334.94)	0.97 (0.95–0.98)	68.06	85.14	0.93
EULAR’19	(10)	4550/6812	94.79 (92.03–96.63)	88.25 (80.88–93.03)	8.07 (4.87–13.38)	0.06 (0.04–0.09)	136.60 (67.28-277.37)	0.97 (0.95–0.98)	57.86	84.59	0.58
**Childhood-onset systemic lupus erythematosus studies**											
ACR’97	(11)	1672/3005	76.18 (69.61–81.70)	95.89 (91.73–98.00)	18.52 (8.83–38.85)	0.25 (0.19–0.32)	74.55 (30.19-184.12)	0.91 (0.88–0.93)	78.50	67.20	0.26
SLICC’12	(10)	1560/2789	93.76 (89.45–96.39)	93.49 (87.68–96.67)	14.41 (7.43–27.94)	0.07 (0.04–0.12)	216.02 (81.96-569.33)	0.98 (0.96–0.99)	69.88	74.59	0.22
EULAR’19	(7)	1359/2399	88.84 (83.73–92.48)	91.62 (83.11–96.04)	10.60 (5.00–22.48)	0.12 (0.08–0.19)	86.96 (30.40-248.79)	0.95 (0.93–0.96)	78.02	84.35	0.15

95% CI, 95% confidence interval; ACR, American College of Rheumatology; EULAR, European Alliance of Associations for Rheumatology; SLICC, Systemic Lupus International Collaborating Clinics.

Meta-regression and subgroup analyses were performed to identify the potential heterogeneity sources in adult-onset and childhood-onset SLE studies. In the meta-regression results for adult-onset and childhood-onset SLE studies, heterogeneity was observed in all included studies (overall *I*^2^ > 50% in meta-regression). However, we identified a few important parameters as potential sources of heterogeneity. Significant parameters in meta-regression for adult-onset SLE studies included the proportion of patients with neurological involvement and the number of ANA-positive patients. Additionally, the number of ANA-positive controls, disease duration of patients, and anti-dsDNA positivity were significant parameters in childhood-onset SLE studies (Supplementary Table 4). Next, we performed subgroup analyses according to the significant parameters in the results of meta-regression and other possible parameters related to heterogeneity. We found that apart from studies with the characteristics of percentage of hemolytic anemia < 20% (the EULAR/ACR 2019 *I*^2^ for sensitivity 0.01 in adult-onset SLE studies), studies carried out in EU countries (the EULAR/ACR 2019 *I*^2^ for sensitivity 0.02 in adult-onset SLE studies), percentage of ANA positivity in controls < 50% (the EULAR/ACR 2019 *I*^2^ for sensitivity 40.14 in childhood-onset studies), renal involvement > 35% (*I*^2^ for specificity: ACR 1997 10.31, SLICC 2012 36.12, EULAR/ACR 2019 46.92 in childhood-onset SLE studies), the other subgroup analyses indicated heterogeneity with *I*^2^ > 50% for sensitivity and specificity. The results for each related parameter are presented in Supplementary Table 5 for adult-onset SLE studies and Supplementary Table 6 for childhood-onset SLE studies.

### 3.4 Comparison of ACR 1997, SLICC 2012, and EULAR/ACR 2019 criteria

We conducted network meta-analysis to compare the performance of ACR 1997, SLICC 2012, and EULAR/ACR 2019. In adult-onset SLE studies, the pooled DOR was as follows: ACR 1997 (18 studies), 53.19 (95% CI 26.76–105.73); SLICC 2012 (16 studies), 121.35 (95% CI 51.55–285.65); EULAR/ACR 2019 (10 studies), and 131.57 (95% CI 61.50–281.47). The SUCRA value was highest for EULAR/ACR 2019 (79.5), followed by SLICC 2012 (46.6) and ACR 1997 (23.8). In childhood-onset SLE studies, the pooled DOR was as follows: ACR 1997 (11 studies), 58.92 (95% CI 28.78–120.60); SLICC 2012 (10 studies), 191.07 (95% CI 76.06–480.01); EULAR/ACR 2019 (7 studies), 87.32 (95% CI 27.81–274.16). The SUCRA value was highest for SLICC 2012 (97.2), followed by EULAR/ACR 2019 (34.0) and ACR 1997 (18.8). As expected, the best set of criteria for diagnosing adult-onset SLE based on the SUCRA value was EULAR/ACR 2019, and the best set of criteria for diagnosing childhood-onset SLE was SLICC 2012.

### 3.5 Publication bias

We used Deeks’ funnel plot to assess the publication bias of all included studies in this meta-analysis. Deeks’ funnel plot for each index test revealed a symmetrical funnel shape (adult-onset SLE studies: ACR 1997, *P* = 0.32, SLICC 2012, *P* = 0.93, EULAR/ACR 2019, *P* = 0.58; childhood-onset SLE studies: ACR 1997, *P* = 0.26, SLICC 2012, *P* = 0.22, EULAR/ACR 2019, *P* = 0.15), indicating that there was no publication bias in this meta-analysis (Supplementary Figure 1).

## 4 Discussion

This study demonstrated that the characteristics of patients included in adult-onset and childhood-onset SLE studies had distinct disease phenotypes, especially the frequency of autoimmune hemolytic anemia and renal involvement. EULAR/ACR 2019, the most recent set of classification criteria, performed better in diagnosing patients with adult-onset SLE than those with childhood-onset SLE. SLICC 2012 was the best criteria for diagnosing patients with childhood-onset SLE.

Patients with adult-onset and childhood-onset SLE have different disease phenotypes. Childhood-onset SLE involves higher rates of renal, neuropsychological, and hematological features than adult-onset SLE (56), leading to a more severe disease course. In line with the findings of this study, patients with childhood-onset SLE tend to have a higher rate of hemolytic anemia and renal involvement than patients with adult-onset SLE. Because there are no diagnostic criteria for SLE, the three classification criteria, ACR 1997, SLICC 2012, and EULAR/ACR 2019, are generally applied when diagnosing SLE. In our analysis, SLICC 2012 in childhood-onset SLE had the highest pooled DOR because it incorporates a greater range of hematological, renal, neurological, and laboratory criteria in SLE diagnosis. Regarding hematological involvement, three features of SLICC 2012, including autoimmune hemolytic anemia, leukopenia or lymphopenia, and thrombocytopenia, are separated into three items; patients with a positive direct Coombs test without evidence of hemolytic anemia are also taken into account (16). In contrast, ACR 1997 combines these three features into one item, and EULAR/ACR 2019 counts only one feature with the highest weighted score in the hematological domain (15, 24, 25). Therefore, patients with SLE who initially present with hematologic involvement can be diagnosed early with SLICC 2012, more so than with the other criteria. Moreover, SLICC 2012 includes a broader clinical neurological spectrum (seizure, psychosis, mononeuritis multiplex, myelitis, peripheral or cranial neuropathy, acute confusion state) than ACR 1997 (seizure, psychosis) and EULAR/ACR 2019 (delirium, psychosis, seizure) (15, 16, 24, 25). For renal involvement, ACR 1997 added proteinuria > 500 mg/24 h or > 3+, or cellular casts to one of eleven criteria, while SLICC 2012 placed proteinuria > 500 mg/24 h or RBC casts to one of eleven clinical criteria. Furthermore, SLICC 2012 allows the patients to be diagnosed with SLE if they have biopsy-proven nephritis compatible with SLE and with positive-ANA or anti-dsDNA antibodies. EULAR/ACR 2019 added renal features to one of seven clinical domains with a weighted value of up to 10 points if patients have class III or IV lupus nephritis, which is higher than other domains. A previous study by Smith et al. revealed that SLICC 2012 was more sensitive than EULAR/ACR 2019 (52). They included SLE with lupus nephritis in their analysis, and some patients were ANA-negative upon initial presentation, resulting in a missed diagnosis if using EULAR/ACR 2019. However, around fifty percent of ANA-negative patients in this cohort became ANA-positive over time, enabling them to meet EULAR/ACR 2019 criteria at their most recent visit (52). Therefore, data on the longitudinal expression of ANA may influence the implementation of classification criteria in which ANA expression is a requirement for entry. In contrast with the Levinsky et al. study, which demonstrated that the sensitivity of EULAR/ACR 2019 was comparable to that of SLICC 2012 in childhood-onset SLE and the specificity of EULAR/ACR 2019 was slightly higher than that of SLICC 2012 criteria (30). In this study, the requirement of a positive ANA as an admission criterion had no effect on the sensitivity of EULAR/ACR 2019 since nearly all patients had a positive ANA. Previous studies have shown us that using ANA as an entry criterion has benefits and limitations. Positive ANA aids in the exclusion of disorders with comparable clinical symptoms to SLE, such as hemolytic uremic syndrome (27); however, obligatory positive ANA may reduce the sensitivity of EULAR/ACR 2019, especially in childhood-onset SLE. A previous study reported that ANA positivity varies with age and is highest at diagnosis in patients aged 14–18 years compared with those aged < 8 years or 8–13 years (52). Patients with childhood-onset SLE can have a substantial genetic contribution to disease pathophysiology without producing autoantibodies. This group of patients can have more severe disease and organ damage and a higher proportion of ANA-negative patients than adult-onset SLE (23). Therefore, EULAR/ACR 2019 may have limited utility for the early diagnosis of childhood-onset SLE with negative ANA.

A recent systematic review and meta-analysis study in patients with childhood-onset SLE showed that EULAR/ACR 2019 was not the best tool for diagnosing childhood-onset SLE because its sensitivity was lower than that of SLICC-2012 and its specificity was lower than the ACR 1997 criteria (28). There are two disparities in the study selection criteria between the present study and the previous systematic review and meta-analysis (28). The prior study included full-text papers and conference abstracts and focused only on childhood-onset SLE. In the present study, we exclusively included full-text articles and focused on adult-onset and childhood-onset SLE. Despite some methodological variations, our results supported those of the previous study (28) regarding the performance of EULAR/ACR 2019 in childhood-onset SLE diagnosis. However, in another aspect of using EULAR/ACR 2019, recent studies have highlighted the important role of the EULAR/ACR 2019 in predicting damage accrual of SLE patients at diagnosis (57, 58), particularly the early damage resulting from disease activity itself rather than cumulative glucocorticoid therapy (58).

The DOR of SLICC 2012 and EULAR/ACR 2019 were more comparable in adult-onset SLE studies than in childhood-onset SLE. This proposed set of classification criteria for SLE was mainly developed and validated using primarily large adult SLE cohorts, and the proportion of ANA positivity increases with age (59); this may contribute to the high performance of EULAR/ACR 2019 in the diagnosis of adult-onset SLE. Because adult-onset and childhood-onset SLE exhibit distinctive characteristics and disease phenotypes, future classification criteria should consider these discrepancies to improve performance. Nevertheless, ACR 1997 had the highest specificity and limited sensitivity in adult-onset and childhood-onset SLE studies overall. Thus, ACR 1997 seems suitable for research that includes only a well-defined subgroup of SLE and might exclude atypical patients.

Concerning subgroup analysis, the results for adult-onset and childhood-onset SLE studies aligned with the pooled data. In adult-onset SLE studies, the DOR of each subgroup was highest in either SLICC 2012 or EULAR/ACR 2019, and the DOR of SLICC 2012 was highest in all subgroups of childhood-onset SLE studies. Furthermore, network meta-analysis supported that EULAR/ACR 2019 performed best in diagnosing adult-onset SLE whereas SLICC 2012 performed best in childhood-onset SLE. Differences in the definition of organ involvement, the extent of clinical and immunologic involvement, laboratory data contained in each set of criteria, and the requirement for entry criteria may all impact the performance of these three criteria.

This study has several limitations. First, most studies unavoidably used a case-control design in patient selection because most included studies were retrospective. The reference standard was based on clinical diagnosis, which was subject to interpretation and varied among rheumatologists. Additionally, most studies did not mention the blinding of clinical diagnosis when applying the classification criteria. Lastly, the results of subgroup analysis are limited owing to the relatively small number of included studies, especially childhood-onset SLE studies. Future studies with prospective study designs are needed. However, in the present study, we revealed important aspects regarding the diagnosis of patients with adult-onset and childhood-onset SLE using the three most common and up-to-date criteria.

## 5 Conclusion

The diagnostic performance of three classification criteria, ACR 1997, SLICC 2012, and EULAR/ACR 2019, differed between adult-onset and childhood-onset SLE. Comparisons among the three classification criteria showed that EULAR/ACR 2019 had the best performance for adult-onset SLE, and SLICC 2012 was best for childhood-onset SLE.

## Data availability statement

The original contributions presented in this study are included in the article/Supplementary material, further inquiries can be directed to the corresponding author.

## Author contributions

BL, SR, and SV contributed to the study conception and design and conducted the data analysis. BL and PC performed the data extraction. BL, PC, SR, and SV contributed to the data interpretation, writing, and critically revising the manuscript. All authors read and approved the final manuscript.

## Supplementary material

Supplementary Figure 1 Deeks’ funnel plots to evaluate the publication bias of all included adult-onset and childhood-onset systemic lupus erythematosus studies.
