# Supplementary material for: Evaluating performance of the 2019 EULAR/ACR, 2012 SLICC, and 1997 ACR criteria for classifying adult-onset and childhood-onset systemic lupus erythematosus: A systematic review and meta-analysis
Source: Front Med (Lausanne). 2022 Dec 22;9:1093213. doi: 10.3389/fmed.2022.1093213 (PMC9813386; doi:10.3389/fmed.2022.1093213)
Supplement: Supplementary file 1 [file Table_1.docx]

**Table S1** QUADAS-2 tool for assessing methodological quality and risk of bias of included studies for this meta-analysis.

|  | **Signaling question** | **Signaling question** | **Signaling question** | **Risk of bias** | **Concerns about**  **applicability** |
| --- | --- | --- | --- | --- | --- |
| **Domain 1: Patient selection** | | | | | |
| **Patient**  **selection** | Was a consecutive or  random sample of  patients enrolled? | Was a case-control  design avoided? | Did the study avoid  inappropriate exclusions? | Could the selection  of patients have  introduced bias? | Are there concerns  that the included  patients and setting  do not match the review  question? |
|  | Yes: If all consecutive or random samples of subjects were enrolled.  No: If subjects were nonrandomly or non-consecutively  selected.  Unclear: If sampling method was unclear. | Yes: If the study was  not a case-control  design.  No: If the study had a  case-control design.  Unclear: If the study  design was unclear. | Yes: If there were no  inappropriate exclusion  criteria.  No: If subjects were  excluded based on  inappropriate criteria such as presence of  anxiety.  Unclear: If selection  criteria were unclear. | Low risk: If all signaling questions answered “yes”  High or unclear risk: If  “No or unclear” was  reported for at least one signaling question. | Low concern: If selected subjects matched the review question and  Inappropriate exclusions  were avoided.  High concern: If selected subjects differed from those  in the review question.  Unclear concern: If there was insufficient information on included subjects and setting. |
| **Domain 2: Index test** | | | | | |
| **Index test** | Were the index test results interpreted without knowledge of the results of the reference standard? | If a threshold was  used, was it pre-specified? |  | Could the conduct  or interpretation of  the index test has  introduced bias? | Are there concerns  that the index test,  its conduct or interpretation  differ from the review  question? |
|  | Yes: If the index test results were interpreted without  knowledge of the  clinical diagnosis.  No: If the index test results  were interpreted with  knowledge of the  clinical diagnosis.  Unclear: If it was unclear whether index test results  were interpreted  independently of  clinical diagnosis. | Yes: If the threshold  for a positive test result  was pre-specified.  No: If the threshold for  a positive test result  was not pre-specified.  Unclear: If this was unclear from the  report. |  | Low risk: If all signaling questions answered “yes”  High or unclear risk: If  “No or unclear” was reported for at least one signaling  question. | Low concern: If the index test was performed as described in the review question.  High concern: If the index test differed from those specified in the review question.  Unclear concern: If there was insufficient information  available. |
| **Domain 3: Reference standard** | | | | | |
| **Reference**  **standard** | Is the reference standard likely to correctly classify the  target condition? | Were the reference  standard results  interpreted without  knowledge of the results of the index test? |  | Could the reference  standard, its conduct,  or its interpretation have introduced bias? | Are there concerns  that the target condition  as defined by the reference standard does not match the review question? |
|  | Yes: If the clinical diagnosis was used.  No: If the clinical diagnosis was not  used.  Unclear: If it was unclear from the report whether  the clinical diagnosis was used. | Yes: If the clinical diagnosis results were interpreted without knowledge of the index test results.  No: If the clinical diagnosis results  were interpreted with  knowledge of the index  test results.  Unclear: If this was not  clear from the report. |  | Low risk: If all signaling questions answered “yes”  High or unclear risk: If  “No or unclear” was reported for at least one signaling  question. | Low concern: If the clinical diagnosis was used.  High concern: If the clinical diagnosis was not used.  Unclear concern: If  insufficient information was provided in the report. |
| **Domain 4: Flow and timing** | | | | | |
| **Flow and**  **timing** | Was there an appropriate interval between the index  test and reference standard? | Did all patients receive  the reference  standard? | Were all patients included in the analysis? | Could the patient flow  have introduced bias? |  |
|  | Yes: If the time between the index and reference tests  were less than six months.  No: If the time between the index and reference tests  were longer than six months.  Unclear: If this was unclear from the report. | Yes: If all eligible  subjects received  the clinical diagnosis.  No: If not all eligible subjects received the clinical diagnosis.  Unclear: If this was not  clear from the report | Yes: If all subjects  recruited to the study with index test results were included in the analysis.  No: If not all recruited  subjects with index test  results were included in  the analysis.  Unclear: If this was  unclear from the report. | Low risk: If all signaling questions answered “yes”  High or unclear risk: If “no or unclear” was reported for at least one signaling  question. |  |
