# Supplementary material for: Evaluating performance of the 2019 EULAR/ACR, 2012 SLICC, and 1997 ACR criteria for classifying adult-onset and childhood-onset systemic lupus erythematosus: A systematic review and meta-analysis
Source: Front Med (Lausanne). 2022 Dec 22;9:1093213. doi: 10.3389/fmed.2022.1093213 (PMC9813386; doi:10.3389/fmed.2022.1093213)
Supplement: Supplementary file 2 [file Table_2.docx]

**Table S2** Characteristics of adult-onset systemic lupus erythematosus studies.

| **First Author** | **Number of centres, setting,**  **country** | **Index test** | **Study design** | **Ethnicity** | **Number** | **%Female** | **Age of onset (years)** | **Age of diagnosis (years)** | **Disease duration (months)** | **%ANA+** | **Inclusion criterion cases** | **Inclusion criterion controls** | **% Of each specified in the control** | SLE characteristics cases (%) |
| --- | --- | --- | --- | --- | --- | --- | --- | --- | --- | --- | --- | --- | --- | --- |
|  |  |  |  | **Case, control** | | | | | | |  |  |  |  |
| Sanchez, 2003 | 1, academic, USA | SLICC'12,  ACR'97 | retrospective | Caucasians 51%, NA; African Americans 47%, NA; others 2%, NA | 267,96 | 90, NA | NA | 44, NA | NA | NA | patients identified with ICD9 code 710.0 and clinical diagnosis of SLE by the rheumatologists | patients identified with ICD9 code 710.0 and no clinical diagnosis of SLE by the rheumatologists | NA | NA |
| Oku, 2017 | 12, tertiary, Japan | SLICC'12  ACR'97 | retrospective | NA | 234, 253  (8 not reach consensus) | 88.5, 81 | NA | 37,53 | NA | 100, 73.1 | each participating institution submitted consecutive data of 20–30 patients with SLE | each participating institution submitted consecutive data of 20–30 patients with non-SLE | RA, PM/DM, UCTD, MCTD, Behcet's disease, vasculitis syndrome, PAPS, SSc, fibromyalgia, SS, psoriasis, sarcoidosis, JIA | anti-dsDNA 79.9%,  APL 22.2%,  low complement 85%, |
| Mosca, 2019 | 7, academic, Asia, Europe, North America, South America, Italy  (Early  SLE of the Italian Society of Rheumatology) | SLICC'12,  ACR'97 | retrospective | Caucasian 54.5%, 89.4%; Asian 29%, 6.2%; African descent 7.7%, 2.6%; American Indian 0.3%, 0%; unknown 6.7%, 1.8% | 389, 227 | 88.9, 96.9 | 31.4, 33.9 | NA | NA | 99.5, 95.1 | possible SLE within the previous  3 years and had a symptom duration <1 y | non-SLE patients were those who were referred during the same period  of time due to suspected SLE | UCTD 59.9%, SS 9.3%,  SSc 4.8%,  primary RP 4.4%, fibromyalgia 3.5%,  ANA-positive  thyroiditis 3.1%, RA 2.6%, MCTD 1.8%, hematologic diseases 0.9%, infections 0.9%, AH 0.4%, psoriatic arthritis  0.4%,  miscellaneous diagnoses  7.9% | malar rash 49.6%,  discoid rash 9.3%, photosensitivity 31.6%, renal 13.1%,  seizure 2.8%,  psychosis 1%,  cognitive impairment 1.5%,  stroke 1%,  TIA 0.3%,  hemolytic anemia 4.6%, leukopenia 16.2%, thrombocytopenia 6.6%,  pleuritis 22.4%,  pericarditis 18.8%,  anti-dsDNA 71.7%,  IgG anticardiolipin Ab 18.1%,  lupus anticoagulant 12.7%  anti-β2GPI 17%  low complement 73.4% |
| Sacre, 2021 | 1, academic, France | EULAR'19,  SLICC'12,  ACR'97 | retrospective | NA | 17, 32 | 100, 53.1 | NA | 32.4, 50.6 | NA | 100, 100 | patients with pericardial  effusion and a final diagnosis of SLE | patients with pericardial  effusion and non-SLE autoimmune diseases or idiopathic pericarditis | PSS 6.3%, UCTD 6.3%, SSc 6.3%, idiopathic pericarditis 81.3% | ACLE 11.8%,  SCLE 5.9%,  pericardial effusion 100%  acute pericarditis 47.1%,  pleural effusion 58.8%  seizure 11.8%,  psychosis 0%,  delirium 5.9%,  leukopenia 35.3%,  thrombocytopenia 29.4%,  autoimmune hemolysis 11.8%,  proteinuria >0.5 g/24 h 64.7%,  LN class II or V 17.6%,  LN class III or IV 35.3%,  anti-dsDNA 76.5%,  APL 52.9%,  low complement 70.6% |
| Amezcua-Guerra, 2015 | 1, academic,  Mexico | EULAR’19,  ACR’97 | retrospective | NA | 100, 100 | 85, 93 | NA | 37.9, 52 | 82.8, 60 | 99, 56 | patients with clinical diagnosis (the same diagnosis for one year) performed  by an experienced rheumatologist at the institute  staff who did not participate in this study | patients with a clinical diagnosis other than SLE | RA 60%,  PSS 13%, fibromyalgia 12%,  SSc 8%, myositis 7% | malar rash 71%,  discoid rash 1%, photosensitivity 81%,  renal 46%,  neurological 12%,  hematological 86%,  hemolytic anemia 3%, leukopenia/lymphopenia 66%,  thrombocytopenia 14%,  serositis 15%,  renal biopsy 39%,  anti-dsDNA 63%,  IgG anticardiolipin Ab 16%,  IgM anticardiolipin Ab 21%,  lupus anticoagulant 11%  IgG anti-β2GPI 10%  IgM anti-β2GPI 17%  low complement 60% |
| Bakula, 2019 | 1, academic,  Croatia | SLICC'12,  ACR'97 | retrospective | NA | 146, 162 | 90.4, 90.7 | NA | 41, NA | 120, NA | 92.5, 85.8 | patients with SLE and regular follow up | SLE-allied conditions and regular follow up | collagenosis, APS, SS, MCTD,  UCTD, DLE, RP, GN, overlap syndrome,  oligoarthritis HLAA1B8DR3 positive, lymphopenia | malar rash 17.8%,  discoid rash 12.3%, photosensitivity 18.5%, renal 12.3%,  neurological 4.1%,  hematological 47.3%,  hemolytic anemia 4.6%, leuko/lymphopenia 43.2%, thrombocytopenia 12.3%,  serositis 6.9%,  anti-dsDNA 70.5%,  APL 44.5%  low complement 52%  DCT 5.5% |
| Jia, 2017 | 1, tertiary,  USA | ACR'97 | retrospective | Non-White 33.4%, 10.7% | 320, 186 | 94.1, 95.2 | NA | 39.8, 44.1 | NA | NA | patients in pure SLE group | patients in MCTD and UCTD group | MCTD 19.9%, UCTD 80.1% | mucosal disease 35%  skin rash 67.5%  hematological 32.5%,  serosal disease 20%,  renal 25%,  neurological 42.5% |
| Assan, 2021 | 1, academic,  France | EULAR'19,  SLICC'12,  ACR'97 | retrospective | NA | 49, 49 | 81.6, 100 | NA | 33, 54 | 170.4, 172.8 | 98, 73.5 | inpatients and outpatients' SLE cases followed in the department of rheumatology | PSS were randomly chosen from the  Paris Sud database and compared in a 1:1 ratio with the SLE group (all patients with PSS fulfilled the pSS ACR/EULAR  2016 criteria) | PSS 100% | ACLE 44.9%,  CCLE 16.3%,  pleurisy 14.3%,  pericarditis 8.2%,  leukopenia 18.4%,  lymphopenia 32.7%,  anti-dsDNA 89.8%,  APL 40.8%,  low C3 26.5%  low C4 49% |
| Gegenava, 2019 | 1, academic,  Netherlands | EULAR'19,  SLICC'12,  ACR'97 | retrospective | NA | 294, 66 | 87.1, 83.3 | NA | 43, 46 | NA | 96.3, 75.8 | patients referred to the NPSLE clinic in the Leiden  University Medical Center and had a clinical diagnosis of SLE | patients without the clinical diagnosis of SLE | SLE-like disease 30.3%, UCTD 18.2%, PSS 16.7%, MCTD 12.1%, others (chilblain LE, chronic discoid lupus, APS, DM, CREST syndrome, JIA, Behcet-like disease, somatoform disorder) 22.7% | malar rash 43.9%,  discoid rash 17.7%, photosensitivity 52.7%,  ACLE or SCLE 50.3%  CCLE 17.7%  renal 29.9%,  proteinuria 22.4%  LN class II or V 10.5%  LN class III or IV 12.6%  neurological 12.6%,  psychosis 4.1%  seizure 9.5%  mononeuritis 1.7%  myelitis 3.1%  neuropathy 3.4%  hematological 49.3%  hemolytic anemia 14.6%, leukopenia 47.6%,  thrombocytopenia 19.7%  serositis 25.9%,  anti-dsDNA 57.7%,  APL 48.6%,  low complement 57.1%,  DCT 7.1% |
| Flynn, 2018 | 1, academic,  Ireland | SLICC'12,  ACR'97 | retrospective | NA | 14, 22 | 94, NA | NA | NA | NA | NA | all patients who attended the cutaneous lupus clinic during the previous four months and biopsy-proven cutaneous lupus labeled as SLE in their medical notes | all patients who attended the cutaneous lupus clinic during the previous four months and had biopsy-proven cutaneous lupus with not labeled as SLE in their medical notes | cutaneous lupus erythematosus | NA |
| Rijnink, 2017 | 1, academic,  Netherlands | SLICC’12,  ACR’97 | retrospective | NA | 117, 32 | 74.4, 34.4 | NA | 32.6, 38.7 | 127.2, 240 | 99.1, 11.5 | renal biopsy showing full house glomerular deposits with clinical SLE | renal biopsy showing full house glomerular deposits without clinical SLE | membranous nephropathy  3.1%,  cancer-associated 9.4%,  IgA nephropathy 12.5%, infection-related glomerulonephritis 6.3%, ANCA–  associated glomerulonephritis 6.3%, idiopathic non–  lupus full house nephropathy 62.5% | malar rash 42.9%,  photosensitivity 22.3%, discoid rash 9.4%,  ACLE or SCLE 56.4%, CCLE 12%  neurological 15.4%, hemolytic anemia 17.1%, lymphopenia/leukopenia 30.8%,  thrombocytopenia 23.9%  serositis 37.6%,  anti-dsDNA 73.4%,  APL 50.6%,  low complement 86.2%,  DCT 33.3% |
| Jin, 2020 | 33, academic, China | EULAR'19,  SLICC'12,  ACR'97 | retrospective | NA | 1,865, 232 | 90.6, 68.1 | 30.2, 32.1 | 31.1, 33.9 | 58.6, 53.6 | NA | SLE patients from the dermatology, rheumatology, and nephrology department with a final diagnosis of SLE | Isolated-CLE | Isolated-CLE 100% | NA |
| Teng, 2020 | 1, academic,  China | EULAR'19,  SLICC'12,  ACR'97 | retrospective | NA | 199, 175 | 91, 78 | NA | 37.7, 53.8 | 10.7, 24.6 | 99.5, 69.7 | new-onset SLE patients were enrolled from among the consecutive  individuals at outpatient clinics or hospitalized patients | clinical  diagnosis other than SLE | RA 39%,  SSc 7%,  PSS 31%,  DM 21%, lymphoma 2% | ACLE 23%,  SCLE/CCLE 24%,  pleural or pericardial effusion 14%  acute pericarditis 2%  renal 31%,  proteinuria >0.5 g/24 h 31%,  LN class II or V 1%  LN class III or IV 2%  psychosis 0.5%  seizure 0.5%  delirium 0.5%  hemolytic anemia 45%, leucopenia 62%, thrombocytopenia 28%,  serositis 16%,  anti-dsDNA 86%,  APL 28.6%,  IgG anticardiolipin Ab 7%,  lupus anticoagulant 21%,  IgG anti-β2GPI 18%,  low complement 80%,  DCT 48% |
| Aringer, 2020 | 21, USA, Canada, Mexico, Austria,  Croatia, France, Germany, Greece, Hungary, Italy, Portugal,  Spain, the UK, Turkey, Hong Kong and Japan  Canada | EULAR'19,  SLICC'12,  ACR'97 | Retrospective  (validation cohort) | White 69%, 80.3%; Hispanic 10.5%, 8.9%;  Black 8%, 2.1%; east Asian 7.6%, 5.9%; south/southeast Asian 3%,1.9%; other 1.9%, 0.9% | 696, 574 | 87.4, 85.4 | NA | 45, 56 | 132, 108 | 99.3, NA | SLE from each center up to 100 cases | controls with conditions mimicking SLE from each center as equal number of cases | SS 21.6%,  SSc 20.9%,  RA 19.2%, PAPS 8.4%, inflammatory myositis 4.7%,  UCTD 3.5%, MCTD 2.6%, membranous nephritis 2.4%, vasculitis 2.3%,  adult onset still's disease 1.9%,  Behcet's disease 1.6%,  psoriatic arthritis 1.6%,  other 5.1% | NA |
| Petri, 2021 | 15, academic, USA | EULAR'19,  SLICC'12,  ACR'97 | retrospective | NA | 293, 423 | NA | NA | NA | NA | NA | clinical diagnosis of SLE patients | non-SLE patients | RA 28.1%, myositis 13%, CCLE 11.8%, UCTD 10.4%, vasculitis 8.7%, APS 7.8%, scleroderma 6.6%, fibromyalgia 5.9%,  SS 3.5%,  rosacea 1.9%,  psoriasis 1.7%, | NA |
| Adamichou, 2020 | 2, academic, Greece | EULAR'19,  SLICC'12,  ACR'97 | retrospective | White, Caucasian 99.3%, 99% | 690, 401 | 92.2, 78.6 | NA | 42, 50 | 48, 47 | 93.6, 39.7 | age of diagnosis ≥16 years, known ANA status, and follow-up ≥6 months to confirm the diagnosis | randomly selected from electronic databased of lupus mimicking rheumatologic disease | RA 30.9%, UCTD 13.2%, SS 10.5%,  SSc 10%,  psoriatic arthritis 6.2%,  vasculitis 5.5%,  Behcet's disease 4.7%,  myositis 4.5%,  FMF 4.2%,  Adult-onset still’s disease 3.2%,  fibromyalgia 3.2%,  PAPS 2.2%,  CCLE 1.5% | malar rash 62.6%,  photosensitivity 70%,  discoid rash 8.7%,  neurological 6.5%,  renal 12.5%,  LN class II or V 3.2%,  LN class III or IV 4.8%  haematological 46.1%,  leukopenia 32.3%,  lymphopenia 7.1%,  thrombocytopenia 15.2%,  hemolytic anemia 2.9%,  serositis 13.6%,  anti-dsDNA 27%,  low C3 or low C4 14.93%,  low C3 and C4 23.3%  APL 19.7%,  DCT 5.1% |
| Dahlstrom, 2019 | 1, academic, Sweden | EULAR proposed,  SLICC'12,  ACR'97 | retrospective | NA | 56, 55 | 78.6, 87.3 | NA | 49.7, 53.5 | NA | 98.2, 89.1 | All had an SLE diagnosis confirmed by one single senior rheumatologist and fulfilled FDP and/or ACR 82 | possible systemic autoimmune disease, including  the presence of any SLE-related autoantibody follow-up≥5 years | PSS 21.8%, UCTD 14.5%, APS 12.7%,  RA 7.3%, fibromyalgia 73%,  arthralgia 5.5%, psoriasis arthritis 3.6%, unspecified arthritis 3.6%, adult-onset still disease 1.8%, polymyositis 1.8%,  SSc with primary biliary cirrhosis 1.8%, MCTD 1.8%, pyogenic arthritis, pyoderma gangrenosum  and acne syndrome 1.8%,  renal infarction 1.8%,  multiple sclerosis 1.8%, palindromic rheumatism  1.8%,  recurrent pleuritis 1.8% | ACLE 26.8%  CCLE 19.6%  photosensitivity 44.6%  pleuritis 25%  pericarditis 12.5%  renal 30.4%,  biopsy-proven LN 26.8%  neurological (ACR-82) 3.6%  neurological (SLICC-12) 5.4%  hemolytic anemia 3.6%, leucopenia 48.2%,  lymphopenia 51.8%  thrombocytopenia 8.9%,  anti-dsDNA 51.8%,  anticardiolipin Ab 10.7%,  lupus anticoagulant 17.9%  anti-β2GPI 16.1%  low complement 53.6%,  DCT 50% |
| Lee, 2020 | 1, academic, Korea | EULAR'19,  SLICC'12,  ACR'97 | retrospective | NA | 335, 337 | 90.1, 86.6 | NA | 32, 48 | NA | 98.8, 72.4 | all SLE patients  followed up by rheumatologists for more than two years. | patients  with a well-established clinical diagnosis  of other rheumatic diseases were  randomly selected | RA 27.3%,  APS 16.9%, MCTD 15.4%, SSc 12.8%, PSS 11.6%, UCTD 8.3%, RA with secondary SS 7.1%,  DM 0.3%, spondyloarthropathy 0.3% | ACLE 63.3%  CCLE 7.5%  photosensitivity 26%  serositis 28.4%  proteinuria 60.9%,  biopsy-proven LN 33.4%  neurological 15.2%  hemolytic anemia 34.3%, leucopenia 64.2%,  lymphopenia 56.7%  thrombocytopenia 39.4%,  anti-dsDNA 89.3%,  anticardiolipin Ab 14.6%,  lupus anticoagulant 25.1%  anti-β2GPI 4.5%  low C3 or low C4 84.7%,  low C3 and low C4 73.4% |

ACR; American College of Rheumatology, AE; autoimmune en­cephalitis, AH; autoimmune hepatitis, AIHA; auto­immune hemolytic anemia, ALPS; acute lymphoproliferative syndrome, Anti-dsDNA, anti-double stranded DNA, ANCA; antineutrophil cytoplasmic antibody, APL; antiphospholipid antibodies , APS; antiphospholipid syndrome, APSGN; acute post-streptococcal glomerulonephritis, ACLE; Acute cutaneous lupus erythematosus, C3; complement component C3, CCLE; chronic cutaneous lupus erythematosus, CREST; Calcinosis-Raynaud phenomenon-Esophageal involvement-Sclerodactyly-Telangiectasia, DCT; direct coombs' test, DLE; discoid lupus erythematosus, DM; dermatomyositis, EULAR; European League Against Rheumatism, FMF; familial Mediterranean fever, GN; glomerulonephritis, HUS; hemolytic uremic syndrome, ICE; International Classification of Diseases, ITP; idiopathic thrombocytopenic purpura, Ig; immunoglobulin, JIA; juvenile idiopathic arthritis, LN; lupus nephritis, MCTD; mixed connective tissue disease, MWS; Muck­le-Wells syndrome, NA; not available, PAN; polyarteritis nodosa, PAPS; primary antiphospholipid syndrome, PM; polymyositis, PSS; primary Sjögren's syndrome, RA; rheumatoid arthritis, RF; rheumatic fever, RP; Raynaud’s phenomenon, SCLE; subacute cutaneous lupus erythematosus, SLICC; Systemic Lupus International Collaborating Clinics, SS; Sjögren's syndrome, SSc; systemic sclerosis, SLE; systemic lupus erythematosus, TIA; transient ischemic attack, UCTD; undifferentiated connective tissue disease, y; year.
