# Supplementary material for: Evaluating performance of the 2019 EULAR/ACR, 2012 SLICC, and 1997 ACR criteria for classifying adult-onset and childhood-onset systemic lupus erythematosus: A systematic review and meta-analysis
Source: Front Med (Lausanne). 2022 Dec 22;9:1093213. doi: 10.3389/fmed.2022.1093213 (PMC9813386; doi:10.3389/fmed.2022.1093213)
Supplement: Supplementary file 3 [file Table_3.docx]

**Table S3** Characteristics of childhood-onset systemic lupus erythematosus studies.

| First Author | Number of centres, setting,  country | Index test | Study design | Ethnicity | Number | %Female | Age of onset (years) | Age of diagnosis (years) | Disease duration (months) | %ANA+ | Inclusion criterion cases | Inclusion criterion controls | % Of each specified in the control | SLE characteristics cases (%) |
| --- | --- | --- | --- | --- | --- | --- | --- | --- | --- | --- | --- | --- | --- | --- |
|  |  |  |  | Case, control | | | | | | |  |  |  |  |
| Fonseca, 2015 | 1, tertiary, Brazil | SLICC'12,  ACR'97 | retrospective | NA | 81, 92 | 85.3, 66.7 | 9.9, 6.6 | 10.4, 7.4 | NA | 93.8, 51.1 | children and adolescents with cSLE followed up for >1  year | other rheumatic diseases | SJIA 40.2%,  JDM 35.9%,  JSS 5.4%,  MCTD 3.3%,  SS 3.3%,  PAPS 3.3%, primary vasculitis 8.7% | malar rash 55.6%,  discoid rash 6.2%, photosensitivity 21%,  renal 44.4%,  neurological 17.3%,  hematological 76.5%,  serositis 33.3%,  anti-dsDNA 53.1%,  APL 25.9%,  low complement 50.6%,  DCT 24.7% |
| Sag, 2014 | 3, academic,  Italy, UK, Turkey | SLICC'12  ACR'97 | retrospective | NA | 154, 123 | 83.1 | NA | 12.7, 8.9 | NA | 96.4, NA | cSLE were younger than 18 years of age at onset | admitted to  the same centers in the same period, ANA included in the diagnostic work-up | primary vasculitis 35%,  JIA 13%,  JDM 6.5%,  unclassified vasculitides 4.1%,  ITP 4.9%,  septic arthritis 3.3%,  HUS 4.1%,  ALPS 1.6%,  FMF 0.8%,  AIHA 0.8%,  MWS 1.6%,  AE 0.8%,  SSc 6.5%,  APSGN 1.6%, MCTD 0.8%,  RF 4.1%,  undifferentiated diagnoses 8.1% | ACLE 60%,  CCLE 7.3%,  renal 43.6%,  neurological 18.2%,  hemolytic anemia 9.1%, leukopenia 29.1%, thrombocytopenia 16.4%,  serositis 10.9%,  anti-dsDNA 74.5%,  APL 40%,  low complement 85.5%,  DCT 27.3% |
| Arango, 2018 | 1, tertiary, Colombia | SLICC'12,  ACR'97 | retrospective | NA | 55, 55 | 84.4, 64.3 | 12.8, 11.1 | NA | NA | 94, 35 | the first month of diagnosis in patients with cSLE | patients with different diagnoses who attended  the pediatric rheumatology clinic during the same period | JIA 43.6%,  JDM 12.7%, autoimmune hematologic disease 10.9%, APS 10.9%, systemic vasculitis 9.1%, overlapping syndrome 7.3%,  poorly differentiated autoimmune disease 3.6%,  AH 1.8% | malar rash 35%,  discoid rash 7%, photosensitivity 11%, proteinuria 42%,  renal cylinders 5%,  seizure 4%,  peripheral neuropathy 2%,  hemolytic anemia 11%, leukopenia 36%, thrombocytopenia 29%,  pleuritis 15%,  pericarditis 5%,  anti-dsDNA 57%,  IgG anticardiolipin Ab 27%,  lupus anticoagulant 24%  low C3 80%,  low C4 69% |
| Osaku, 2018 | 1, tertiary, Brazil | SLICC'12,  ACR'97 | cross-sectional | NA | 23, 24 | 100, 66.7 | 11.5, 7.9 | NA | NA | 100, 41.7 | patients up to 18 years old, followed in service, with less than one month after diagnosis | patients up to 16 years old, with less than one month after diagnosis | JIA 100% | ACLE 47.8%,  CCLE 4.3%,  renal 60.9%,  neurological 4.3%,  hematological 69.6%  hemolytic anemia 30.4%, leukopenia/lymphopenia 52.2%,  thrombocytopenia 17.4%,  serositis 43.4,  anti-dsDNA 52.2%,  APL 26.1%,  low complement 60.9%,  DCT 21.7% |
| Aljaberi,2021 | 1, tertiary,  USA | EULAR'19,  ACR'97 | retrospective | White 57.1%, 82.9% | 112, 105 | 81, 71 | NA | 15, 10 | NA | 96, 71 | age ≤21 years at the time of initial diagnosis of  SLE by a pediatric rheumatologist | JDM, JSSc according to ICD-10 | JDM 66%,  JSSc 34% | cutaneous 72%,  renal 28%,  neurological 12%,  hematological 61%  serositis 15%,  APL 41%,  low complement 69% |
| Batu, 2021 | 3, academic,  Turkey | EULAR'19,  SLICC'12,  ACR'97 | cohort | NA | 262, 174 | 80.9, 60.9 | 12.6, 9 | 13.3, 9.3 | NA | 98.9, 73 | patients were enrolled at the pediatric rheumatology units, diagnosed before 18 years of age | patients with ANA test results available  (positive or negative) and admitted to Hacettepe University | PAN,  Behçet disease, IgA vasculitis, JDM,  JIA | malar rash 48.9%,  discoid rash 3.8%, photosensitivity 26.7%, proteinuria 26.3%,  urinary casts 14.9%, biopsy-proven LN 37%,  seizure 5.7%,  peripheral neuropathy 1.9%,  acute confusional state 1.9%  delirium 0.8%,  psychosis 1.5%,  hematologic 55.3%,  hemolytic anemia 24.8%, leukopenia 22.9%, thrombocytopenia 25.6%,  pleuritis 2.3%,  pericarditis 5%,  anti-dsDNA 62.6%,  anticardiolipin Ab 22.5%,  antiβ2-GPI 18.9%,  lupus anticoagulant 16.8%  low C3 60.7%,  low C4 69.5%  DCT 39% |
| Levinsky, 2021 | 3, tertiary,  Israel | EULAR'19,  SLICC'12,  ACR'97 | retrospective | Caucasian 92.8%, 88.5%; Europe 19.6%, 25.7%; middle east 39.3%, 31.9%; mixed 18.75%, 23.9%;  other/  unknown 22.3%, 18.6% | 112, 113 | 83, 68.1 | 13, 10.1 | NA | NA | 98.2, 61.1 | patients diagnosed with cSLE between  January 2008 and March 2018, with a clinical follow-up  of at least 2 years | patients with conditions mimicking  SLE, regardless of their specific clinical or immunological  manifestations | ITP 23%,  Poly JIA 17.7% | ACLE 49%,  SCLE/discoid rash 6%, proteinuria >0.5 g/24 h 4%, LN class II or V 13%,  LN class III or IV 29%,  seizure 0%,  delirium 0.9%,  psychosis 0%,  hemolytic anemia 32%, leukopenia 46%, thrombocytopenia 30%,  pleural/pericardial effusion 8%,  pericarditis 5%  low complement 64%,  APL 45%, |
| Smith, 2021 | 22, tertiary,  UK, | EULAR'19,  SLICC'12,  ACR'97 | cohort | White; Caucasian 51%, NA,  Black; African/  Caribbean 15%, NA,  South Asian 29%, NA,  other 4%, NA | 482, 129 | 83.3, 71.3 | NA | 12.8, 11 | 39, NA | cases: <8 y 88%,  8-14 y 93%,  14-18 y 95% vs. controls 100% | patients with (1) had data collected between July 2016 and January 2019, (2) an ACR-1997  score of 2 at inclusion, and (3) aged <18 years at the time of recruitment | unselected ANA-positive control cohort (titer  of ≥1:80) over 12 months period | NA | malar rash 62%,  discoid rash 10%,  photosensitivity 25%, neurological 6%,  renal 36%,  hemolytic anemia 24%,  leukopenia 26%,  thrombocytopenia 14%, serositis 17%,  anti-dsDNA 69%,  APL 22%,  low complement 41% |
| Fonseca, 2019 | 1, academic,  Brazil | EULAR'19,  SLICC'12,  ACR'97 | retrospective | NA | 122, 89 | 82.8, 75.3 | 10.3, 9 | 10.6, 9.5 | 72, 72 | 100, 100 | SLE or other rheumatic diseases with ANA ≥1: 80 serum dilution | patients without the clinical diagnosis of SLE | SJIA 9%,  JDM 38.2%, JSSc 11.2%, MCTD 15.7%,  SS 13.5%,  PAPS 3.4%, primary vasculitis 9% | ACLE 49.2%,  CCLE 10.7%,  renal 27%,  neurological 8.2%,  hemolytic anemia 36.1%, leukopenia/lymphopenia 32%,  thrombocytopenia 14.8%  serositis 24.6%,  anti-dsDNA 37.7%,  APL 24.6%,  low complement 56.6%,  DCT 27.9% |
| Ma, 2020 | 1, tertiary,  USA | EULAR'19,  SLICC'12,  ACR'97 | retrospective | African American 26.3%, NA,  white 27%, NA, Asian 22.4%, NA,  Hispanic 23.7%, NA  other 23%, NA,  unknown 1.3%, NA | 156, 379 | 82.7, NA | NA | 13.1, NA | 49.7, NA | 100, 31.3 | patients with SLE < 19 years who were diagnosed at this institute | ANA  positivity who did not fulfill the diagnosis of SLE or diagnosed with another autoimmune disease | JIA 58%,  linear scleroderma 3.1%,  JSS 5.4%,  JDM 2.3% | ACLE 39.7%,  CCLE 4.5%,  renal 25%,  neurological 5.1%,  hematological 66.7%,  serositis 10.9%,  anti-dsDNA 89.1%,  APL 52.6%,  low complement 82.1%,  DCT 16.7% |
| Abdwani, 2021 | 2, tertiary,  Oman | EULAR'19,  SLICC'12,  ACR'97 | retrospective | - | 113, 51 | 71, 84 | 7.3, 5 | 13.6, 10.9 | 73.2, 68.4 | 92, 100 | cSLE, disease  onset before 13 years of age | other rheumatic diseases with  ANA positivity at ≥1:80 serum dilution, follow up at least 1 year | JIA 88.2%, systemic vasculitis 11.8%,  MCTD 3.9% | malar rash 29%,  discoid 6.2%, photosensitivity 11%,  proteinuria 45%,  cellular cast 11%,  neurological 9.7%, hemolytic anemia 27%, leukopenia 44%, thrombocytopenia 16%,  serositis 15%,  anti-dsDNA 69%,  APL 23%,  low complement 25%,  DCT 42% |

ACR; American College of Rheumatology, AE; autoimmune en­cephalitis, AH; autoimmune hepatitis, AIHA; auto­immune hemolytic anemia, ALPS; acute lymphoproliferative syndrome, Anti-dsDNA,

anti-double stranded DNA, APL; antiphospholipid antibodies , APS; antiphospholipid syndrome, APSGN; acute post-streptococcal glomerulonephritis, ACLE; Acute cutaneous lupus erythematosus, C3; complement component C3, CCLE; chronic cutaneous lupus erythematosus, DCT; direct coombs' test, DLE; discoid lupus erythematosus, EULAR; European League Against Rheumatism, FMF; familial Mediterranean fever, GN; glomerulonephritis, HUS; hemolytic uremic syndrome, ITP; idiopathic thrombocytopenic purpura, Ig; immunoglobulin, JIA; juvenile idiopathic arthritis, JDM; juvenile dermatomyositis, cSLE; childhood-onset systemic lupus erythematosus, JSS; juvenile Sjögren's syndrome, JSSc; juvenile systemic sclerosis, LN; lupus nephritis, MCTD; mixed connective tissue disease, MWS; Muck­le-Wells syndrome, NA; not available, PAN; polyarteritis nodosa, PAPS; primary antiphospholipid syndrome, PSS; primary Sjögren's syndrome, RF; rheumatic fever, RP; Raynaud's phenomenon, SLICC; Systemic Lupus International Collaborating Clinics, SS; Sjögren's syndrome, SSc; systemic sclerosis, SJIA; systemic juvenile idiopathic arthritis, UCTD; undifferentiated connective tissue disease, y; year.
