# Supplementary material for: Evaluating performance of the 2019 EULAR/ACR, 2012 SLICC, and 1997 ACR criteria for classifying adult-onset and childhood-onset systemic lupus erythematosus: A systematic review and meta-analysis
Source: Front Med (Lausanne). 2022 Dec 22;9:1093213. doi: 10.3389/fmed.2022.1093213 (PMC9813386; doi:10.3389/fmed.2022.1093213)
Supplement: Supplementary file 4 [file Table_4.docx]

**Table S4** Meta-regression analysis in adult-onset and childhood-onset systemic lupus erythematosus studies.

| **Parameters** | **Index test** | **Coefficient** | **Standard error** | **Z value** | **P value** | **95% Confidence Interval** | **Heterogeneity: I^2^** |
| --- | --- | --- | --- | --- | --- | --- | --- |
| **Adult-onset systemic lupus erythematosus studies** | | | | | | | |
| Number of female patients | ACR’97 | -0.10 | 0.07 | -1.46 | 0.14 | -0.23 to 0.03 | 93.64 |
|  | SLICC’12 | -0.13 | 0.09 | -1.42 | 0.16 | -0.30 to 0.05 | 94.52 |
|  | EULAR’19 | 0.05 | 0.09 | 0.60 | 0.55 | -0.12 to 0.23 | 91.97 |
| Number of female controls | ACR’97 | -0.04 | 0.03 | -1.46 | 0.15 | -0.10 to 0.01 | 95.51 |
|  | SLICC’12 | -0.04 | 0.03 | -1.27 | 0.21 | -0.10 to 0.02 | 93.88 |
|  | EULAR’19 | 0.03 | 0.04 | 0.73 | 0.46 | -0.05 to 0.12 | 89.86 |
| Disease duration of patients | ACR’97 | 0.00 | 0.01 | 0.49 | 0.62 | -0.01 to 0.02 | 86.22 |
|  | SLICC’12 | 0.00 | 0.01 | 0.46 | 0.65 | -0.02 to 0.03 | 96.21 |
|  | EULAR’19 | 0.01 | 0.01 | 0.82 | 0.41 | -0.01 to 0.03 | 91.68 |
| Patients ANA positivity | ACR’97 | 0.16 | 0.13 | 1.27 | 0.21 | -0.09 to 0.40 | 84.05 |
|  | SLICC’12 | 0.41 | 0.21 | 1.99 | 0.046* | 0.01 to 0.82 | 94.32 |
|  | EULAR’19 | 0.12 | 0.27 | 0.45 | 0.65 | -0.40 to 0.64 | 90.13 |
| Controls ANA positivity | ACR’97 | -0.03 | 0.02 | -1.91 | 0.06 | -0.07 to 0.00 | 82.83 |
|  | SLICC’12 | -0.04 | 0.02 | -1.69 | 0.09 | -0.09 to 0.01 | 92.17 |
|  | EULAR’19 | -0.03 | 0.04 | -0.76 | 0.45 | -0.10 to 0.04 | 88.13 |
| <35% Renal involvement | ACR’97 | -0.93 | 0.73 | -1.27 | 0.20 | -2.36 to 0.50 | 95.28 |
|  | SLICC’12 | -1.50 | 0.77 | -1.94 | 0.05 | -3.01 to 0.02 | 92.73 |
|  | EULAR’19 | -0.25 | 0.80 | -0.31 | 0.76 | -1.82 to 1.33 | 91.13 |
| ≥5% Neurological involvement | ACR’97 | 1.73 | 0.72 | 2.40 | 0.02* | 0.32 to 3.14 | 94.85 |
|  | SLICC’12 | 1.43 | 0.85 | 1.67 | 0.09 | -0.25 to 3.10 | 93.93 |
|  | EULAR’19 | -0.47 | 0.80 | -0.59 | 0.55 | -2.04 to 1.09 | 91.21 |
| ≥50% Anti-dsDNA positivity | ACR’97 | 1.26 | 0.72 | 1.75 | 0.08 | -0.15 to 2.66 | 95.30 |
|  | SLICC’12 | 0.12 | 0.77 | 0.15 | 0.88 | -1.40 to 1.63 | 92.11 |
|  | EULAR’19 | -0.05 | 0.79 | -0.06 | 0.95 | -1.59 to 1.50 | 91.16 |
| <20% Hemolytic anemia | ACR’97 | 0.66 | 0.76 | 0.87 | 0.39 | -0.83 to 2.15 | 95.71 |
|  | SLICC’12 | -0.80 | 0.74 | -1.07 | 0.28 | -2.26 to 0.66 | 92.07 |
|  | EULAR’19 | -1.06 | 0.80 | -1.32 | 0.19 | -2.64 to 0.51 | 90.76 |
| EU country | ACR’97 | 0.24 | 0.76 | 0.31 | 0.76 | -1.25 to 1.72 | 95.66 |
|  | SLICC’12 | -0.80 | 0.81 | -0.98 | 0.33 | -2.40 to 0.80 | 93.47 |
|  | EULAR’19 | -0.53 | 0.79 | -0.67 | 0.50 | -2.08 to 1.02 | 90.92 |
| **Childhood-onset systemic lupus erythematosus studies** | | | | | | | |
| Number of female patients | ACR’97 | 0.07 | 0.06 | 1.13 | 0.26 | -0.05 to 0.20 | 76.60 |
|  | SLICC’12 | 0.06 | 0.08 | 0.81 | 0.42 | -0.09 to 0.22 | 80.03 |
|  | EULAR’19 | 0.05 | 0.13 | 0.36 | 0.72 | -0.21 to 0.31 | 91.05 |
| Number of female controls | ACR’97 | -0.06 | 0.05 | -1.04 | 0.30 | -0.16 to 0.05 | 70.05 |
|  | SLICC’12 | -0.04 | 0.05 | -0.78 | 0.44 | -0.13 to 0.06 | 61.44 |
|  | EULAR’19 | -0.03 | 0.05 | -0.65 | 0.51 | -0.14 to 0.07 | 77.44 |
| Disease duration of patients | ACR’97 | -0.11 | 0.05 | -2.03 | 0.04* | -0.21 to 0.00 | 76.60 |
|  | SLICC’12 | -0.09 | 0.06 | -1.50 | 0.13 | -0.21 to 0.03 | 86.80 |
|  | EULAR’19 | -0.07 | 0.07 | -1.13 | 0.26 | -0.20 to 0.05 | 92.94 |
| Patients ANA positivity | ACR’97 | -0.06 | 0.12 | -0.48 | 0.63 | -0.30 to 0.18 | 74.59 |
|  | SLICC’12 | 0.09 | 0.14 | 0.63 | 0.53 | -0.19 to 0.38 | 79.83 |
|  | EULAR’19 | 0.07 | 0.18 | 0.39 | 0.69 | -0.28 to 0.43 | 91.02 |
| Controls ANA positivity | ACR’97 | -0.03 | 0.01 | -1.93 | 0.05 | -0.06 to 0.00 | 72.48 |
|  | SLICC’12 | -0.02 | 0.02 | -1.36 | 0.17 | -0.06 to 0.01 | 79.96 |
|  | EULAR’19 | -0.04 | 0.02 | -2.42 | 0.02* | -0.08 to -0.01 | 85.02 |
| ≥35% Renal involvement | ACR’97 | 0.01 | 0.85 | 0.02 | 0.99 | -1.65 to 1.68 | 77.62 |
|  | SLICC’12 | -1.54 | 1.09 | -1.42 | 0.16 | -3.67 to 0.59 | 78.87 |
|  | EULAR’19 | -0.89 | 1.12 | -0.80 | 0.42 | -3.08 to 1.29 | 90.88 |
| ≥5% Neurological involvement | ACR’97 | -0.39 | 0.83 | -0.47 | 0.64 | -2.01 to 1.23 | 77.35 |
|  | SLICC’12 | 0.92 | 0.95 | 0.97 | 0.33 | -0.93 to 2.78 | 78.69 |
|  | EULAR’19 | -0.01 | 1.54 | -0.01 | 1.00 | -3.03 to 3.01 | 90.95 |
| ≥50% Anti-dsDNA positivity | ACR’97 | 1.54 | 0.54 | 2.88 | 0.00* | 0.49 to 2.59 | 58.52 |
|  | SLICC’12 | 1.68 | 0.70 | 2.39 | 0.02* | 0.30-3.05 | 65.97 |
|  | EULAR’19 | 1.91 | 0.86 | 2.22 | 0.03* | 0.22-3.60 | 84.34 |
| ≥50% hemolytic anemia | ACR’97 | -0.09 | 0.92 | -0.10 | 0.92 | -1.89 to 1.70 | 77.81 |
|  | SLICC’12 | 0.28 | 1.06 | 0.26 | 0.79 | -1.81-2.36 | 80.35 |
|  | EULAR’19 | NA | NA | NA | NA | NA | NA |

*P value <0.05 was set as significance, ACR; American College of Rheumatology, SLICC; Systemic Lupus International Collaborating Clinics, EULAR; European League Against Rheumatism, EU; European Union, ANA; antinuclear antibody, Anti-dsDNA; Anti double-stranded DNA, NA; not applicable
