# Supplementary material for: Evaluating performance of the 2019 EULAR/ACR, 2012 SLICC, and 1997 ACR criteria for classifying adult-onset and childhood-onset systemic lupus erythematosus: A systematic review and meta-analysis
Source: Front Med (Lausanne). 2022 Dec 22;9:1093213. doi: 10.3389/fmed.2022.1093213 (PMC9813386; doi:10.3389/fmed.2022.1093213)
Supplement: Supplementary file 5 [file Table_5.docx]

**Table S5** Results of subgroup analyses in adult-onset systemic lupus erythematosus studies.

| **Subgroup** | **Index test** | **Included studies** | **TP** | **FP** | **FN** | **TN** | **Pooled sensitivity (%) (95% CI)** | **Pooled specificity (%) (95% CI)** | **Pooled positive likelihood ratio (95% CI)** | **Pooled negative likelihood ratio (95% CI)** | **Pooled diagnostic ratio**  **(95% CI)** | **The area under**  **the curve**  **(95% CI)** | **Heterogeneity: I^2^** | | **P value of Deeks** |
| --- | --- | --- | --- | --- | --- | --- | --- | --- | --- | --- | --- | --- | --- | --- | --- |
|  |  |  |  |  |  |  |  |  |  |  |  |  | **Sensitivity (%)** | **Specificity (%)** |  |
| Disease duration less than five years | ACR’97 | Mosca, 2019 | 257 | 19 | 132 | 208 | 78.12 (54.44-91.43) | 92.72 (84.79-96.68) | 10.74 (5.64-20.43) | 0.24 (0.10-0.54) | 45.50  (19.18-107.92) | 0.94  (0.92-0.96) | 97.99 | 90.42 | 0.75 |
|  |  | Bakula, 2019 | 32 | 3 | 114 | 159 |  |  |  |  |  |  |  |  |  |
|  |  | Jin, 2020 | 1697 | 73 | 168 | 159 |  |  |  |  |  |  |  |  |  |
|  |  | Teng, 2020 | 150 | 7 | 49 | 168 |  |  |  |  |  |  |  |  |  |
|  |  | Adamichou, 2020 | 591 | 28 | 99 | 273 |  |  |  |  |  |  |  |  |  |
|  |  | Lee, 2020 | 320 | 21 | 15 | 316 |  |  |  |  |  |  |  |  |  |
|  | SLICC’12 | Mosca, 2019 | 325 | 40 | 64 | 187 | 93.17  (84.29-97.19) | 81.88  (67.72-90.68) | 5.14  (2.71-9.77) | 0.08  (0.03-0.21) | 61.60  (16.75-226.59) | 0.94  (0.92-0.96) | 93.43 | 95.60 | 0.21 |
|  |  | Bakula, 2019 | 108 | 67 | 38 | 95 |  |  |  |  |  |  |  |  |  |
|  |  | Jin, 2020 | 1833 | 101 | 32 | 131 |  |  |  |  |  |  |  |  |  |
|  |  | Teng, 2020 | 183 | 28 | 16 | 147 |  |  |  |  |  |  |  |  |  |
|  |  | Adamichou, 2020 | 630 | 25 | 60 | 376 |  |  |  |  |  |  |  |  |  |
|  |  | Lee, 2020 | 330 | 25 | 5 | 312 |  |  |  |  |  |  |  |  |  |
|  | EULAR’19 | Jin, 2020 | 1805 | 85 | 60 | 147 | 95.40  (91.63-97.51) | 89.86  (74.85-96.34) | 9.40  (3.60-24.57) | 0.05  (0.03-0.09) | 183.62  (81.37-414.35) | 0.98  (0.96-0.99) | 83.88 | 94.38 | 0.82 |
|  |  | Teng, 2020 | 192 | 17 | 7 | 158 |  |  |  |  |  |  |  |  |  |
|  |  | Adamichou, 2020 | 611 | 11 | 79 | 390 |  |  |  |  |  |  |  |  |  |
|  |  | Lee, 2020 | 327 | 29 | 8 | 308 |  |  |  |  |  |  |  |  |  |
| EU countries | ACR’97 | Sacre, 2021 | 16 | 0 | 1 | 32 | 82.55  (66.22-91.94) | 94.39 (86.91-97.71) | 14.72 (6.24-34.73) | 0.18 (0.09-0.38) | 79.60 (25.54-248.12) | 0.96 (0.94-0.97) | 83.29 | 61.87 | 0.44 |
|  |  | Bakula, 2019 | 32 | 3 | 114 | 159 |  |  |  |  |  |  |  |  |  |
|  |  | Assan, 2021 | 38 | 1 | 11 | 48 |  |  |  |  |  |  |  |  |  |
|  |  | Gegenava, 2019 | 261 | 7 | 33 | 59 |  |  |  |  |  |  |  |  |  |
|  |  | Flynn, 2018 | 12 | 5 | 2 | 17 |  |  |  |  |  |  |  |  |  |
|  |  | Rijnink, 2017 | 110 | 0 | 7 | 32 |  |  |  |  |  |  |  |  |  |
|  |  | Adamichou, 2020 | 591 | 28 | 99 | 273 |  |  |  |  |  |  |  |  |  |
|  |  | Dahlstrom, 2019 | 46 | 10 | 10 | 45 |  |  |  |  |  |  |  |  |  |
|  | SLICC’12 | Sacre, 2021 | 16 | 0 | 1 | 32 | 95.19  (85.75-98.49) | 86.40  (75.04-93.06) | 7.00  (3.58-13.67) | 0.06  (0.02-0.18) | 125.67  (24.78-637.37) | 0.96  (0.94-0.97) | 60.21 | 77.89 | 0.82 |
|  |  | Bakula, 2019 | 108 | 67 | 38 | 95 |  |  |  |  |  |  |  |  |  |
|  |  | Assan, 2021 | 48 | 4 | 1 | 45 |  |  |  |  |  |  |  |  |  |
|  |  | Gegenava, 2019 | 249 | 16 | 45 | 50 |  |  |  |  |  |  |  |  |  |
|  |  | Flynn, 2018 | 12 | 5 | 2 | 17 |  |  |  |  |  |  |  |  |  |
|  |  | Rijnink, 2017 | 117 | 3 | 0 | 29 |  |  |  |  |  |  |  |  |  |
|  |  | Adamichou, 2020 | 630 | 25 | 60 | 376 |  |  |  |  |  |  |  |  |  |
|  |  | Dahlstrom, 2019 | 56 | 14 | 0 | 41 |  |  |  |  |  |  |  |  |  |
|  | EULAR’19 | Sacre, 2021 | 17 | 5 | 0 | 27 | 89.00 (87.00-91.00) | 89.00 (75.00-96.00) | 8.20 (3.40-19.70) | 0.12 (0.10-0.15) | 66.00 (24.00-182.00) | 0.90 (0.87-0.92) | 0.02 | 79.22 | 0.60 |
|  |  | Assan, 2021 | 48 | 2 | 1 | 47 |  |  |  |  |  |  |  |  |  |
|  |  | Gegenava, 2019 | 257 | 17 | 37 | 49 |  |  |  |  |  |  |  |  |  |
|  |  | Adamichou, 2020 | 611 | 11 | 79 | 390 |  |  |  |  |  |  |  |  |  |
|  |  | Dahlstrom, 2019 | 52 | 15 | 4 | 40 |  |  |  |  |  |  |  |  |  |
| Non-EU countries | ACR’97 | Sanchez, 2003 | 231 | 27 | 36 | 69 | 87.49  (78.41-93.09) | 89.57  (78.16-95.37) | 8.39  (3.68-19.12) | 0.14  (0.08-0.26) | 60.07  (16.33-220.91) | 0.94  (0.92-0.96) | 94.38 | 93.07 | 0.24 |
|  |  | Oku, 2017 | 206 | 38 | 28 | 215 |  |  |  |  |  |  |  |  |  |
|  |  | Amezcua-Guerra, 2015 | 97 | 1 | 3 | 99 |  |  |  |  |  |  |  |  |  |
|  |  | Jia, 2017 | 365 | 102 | 263 | 206 |  |  |  |  |  |  |  |  |  |
|  |  | Jin, 2020 | 1697 | 73 | 168 | 159 |  |  |  |  |  |  |  |  |  |
|  |  | Teng, 2020 | 150 | 7 | 49 | 168 |  |  |  |  |  |  |  |  |  |
|  |  | Petri, 2021 | 290 | 15 | 59 | 326 |  |  |  |  |  |  |  |  |  |
|  |  | Lee, 2020 | 320 | 21 | 15 | 316 |  |  |  |  |  |  |  |  |  |
|  | SLICC’12 | Oku, 2017 | 232 | 50 | 2 | 203 | 97.15 (94.60-98.52) | 87.17 (72.52-94.59) | 7.57 (3.35-17.12) | 0.03 (0.02-0.06) | 231.84 (98.00-548.47) | 0.98 (0.97-0.99) | 73.28 | 94.44 | 0.55 |
|  |  | Amezcua-Guerra, 2015 | 92 | 1 | 8 | 99 |  |  |  |  |  |  |  |  |  |
|  |  | Jin, 2020 | 1833 | 101 | 32 | 131 |  |  |  |  |  |  |  |  |  |
|  |  | Teng, 2020 | 183 | 28 | 16 | 147 |  |  |  |  |  |  |  |  |  |
|  |  | Petri, 2021 | 340 | 53 | 9 | 288 |  |  |  |  |  |  |  |  |  |
|  |  | Lee, 2020 | 330 | 25 | 5 | 312 |  |  |  |  |  |  |  |  |  |
|  | EULAR’19 | Jin, 2020 | 1805 | 85 | 60 | 147 | 95.89 (93.06-97.60) | 85.73 (74.24-92.61) | 6.72 (3.59-12.58) | 0.05 (0.03-0.08) | 140.35 (59.86-329.05) | 0.97 (0.96-0.99) | 74.92 | 92.93 | 0.49 |
|  |  | Teng, 2020 | 192 | 17 | 7 | 158 |  |  |  |  |  |  |  |  |  |
|  |  | Petri, 2021 | 317 | 39 | 32 | 302 |  |  |  |  |  |  |  |  |  |
|  |  | Lee, 2020 | 327 | 29 | 8 | 308 |  |  |  |  |  |  |  |  |  |
| ≥50% ANA+ in the control group | ACR’97 | Oku, 2017 | 206 | 38 | 28 | 215 | 83.60 (69.11-92.07) | 94.71 (90.40-97.15) | 15.81 (8.57-29.14) | 0.17 (0.09-0.34) | 91.29 (33.64-247.73) | 0.96 (0.94-0.98) | 89.81 | 69.01 | 0.65 |
|  |  | Mosca, 2019 | 257 | 19 | 132 | 208 |  |  |  |  |  |  |  |  |  |
|  |  | Sacre, 2021 | 16 | 0 | 1 | 32 |  |  |  |  |  |  |  |  |  |
|  |  | Amezcua-Guerra, 2015 | 97 | 1 | 3 | 99 |  |  |  |  |  |  |  |  |  |
|  |  | Bakula, 2019 | 32 | 3 | 114 | 159 |  |  |  |  |  |  |  |  |  |
|  |  | Assan, 2021 | 38 | 1 | 11 | 48 |  |  |  |  |  |  |  |  |  |
|  |  | Gegenava, 2019 | 261 | 7 | 33 | 59 |  |  |  |  |  |  |  |  |  |
|  |  | Teng, 2020 | 150 | 7 | 49 | 168 |  |  |  |  |  |  |  |  |  |
|  |  | Dahlstrom, 2019 | 46 | 10 | 10 | 45 |  |  |  |  |  |  |  |  |  |
|  |  | Lee, 2020 | 320 | 21 | 15 | 316 |  |  |  |  |  |  |  |  |  |
|  | SLICC’12 | Oku, 2017 | 232 | 50 | 2 | 203 | 94.88 (88.68-97.78) | 87.44 (77.45-93.38) | 7.55 (4.02-14.18) | 0.06 (0.03-0.14) | 129.05 (37.37-445.59) | NA | 73.38 | 86.57 | 0.93 |
|  |  | Mosca, 2019 | 325 | 40 | 64 | 187 |  |  |  |  |  |  |  |  |  |
|  |  | Sacre, 2021 | 16 | 0 | 1 | 32 |  |  |  |  |  |  |  |  |  |
|  |  | Amezcua-Guerra, 2015 | 92 | 1 | 8 | 99 |  |  |  |  |  |  |  |  |  |
|  |  | Bakula, 2019 | 108 | 67 | 38 | 95 |  |  |  |  |  |  |  |  |  |
|  |  | Assan, 2021 | 48 | 4 | 1 | 45 |  |  |  |  |  |  |  |  |  |
|  |  | Gegenava, 2019 | 249 | 16 | 45 | 50 |  |  |  |  |  |  |  |  |  |
|  |  | Teng, 2020 | 183 | 28 | 16 | 147 |  |  |  |  |  |  |  |  |  |
|  |  | Dahlstrom, 2019 | 56 | 14 | 0 | 41 |  |  |  |  |  |  |  |  |  |
|  |  | Lee, 2020 | 330 | 25 | 5 | 312 |  |  |  |  |  |  |  |  |  |
|  | EULAR’19 | Sacre, 2021 | 17 | 5 | 0 | 27 | 96.00 (91.00-98.00) | 87.00 (79.00-92.00) | 7.30 (4.30-12.20) | 0.05 (0.02-0.11) | 142.00 (42.00-483.00) | 0.97 (0.95-0.98) | 50.18 | 70.63 | 0.50 |
|  |  | Assan, 2021 | 48 | 2 | 1 | 47 |  |  |  |  |  |  |  |  |  |
|  |  | Gegenava, 2019 | 257 | 17 | 37 | 49 |  |  |  |  |  |  |  |  |  |
|  |  | Teng, 2020 | 192 | 17 | 7 | 158 |  |  |  |  |  |  |  |  |  |
|  |  | Dahlstrom, 2019 | 52 | 15 | 4 | 40 |  |  |  |  |  |  |  |  |  |
|  |  | Lee, 2020 | 327 | 29 | 8 | 308 |  |  |  |  |  |  |  |  |  |
| <35% Renal involvement | ACR’97 | Mosca, 2019 | 257 | 19 | 132 | 208 | 76.16 (57.80-88.16) | 91.24 (84.41-95.25) | 8.70 (4.81-15.74) | 0.26 (0.14-0.49) | 33.29 (12.66-87.55) | 0.93 (0.90-0.95) | 96.79 | 85.80 | 0.84 |
|  |  | Bakula, 2019 | 32 | 3 | 114 | 159 |  |  |  |  |  |  |  |  |  |
|  |  | Jia, 2017 | 365 | 102 | 263 | 206 |  |  |  |  |  |  |  |  |  |
|  |  | Gegenava, 2019 | 261 | 7 | 33 | 59 |  |  |  |  |  |  |  |  |  |
|  |  | Teng, 2020 | 150 | 7 | 49 | 168 |  |  |  |  |  |  |  |  |  |
|  |  | Adamichou, 2020 | 591 | 28 | 99 | 273 |  |  |  |  |  |  |  |  |  |
|  |  | Dahlstrom, 2019 | 46 | 10 | 10 | 45 |  |  |  |  |  |  |  |  |  |
|  |  | Lee, 2020 | 320 | 21 | 15 | 316 |  |  |  |  |  |  |  |  |  |
|  | SLICC’12 | Mosca, 2019 | 325 | 40 | 64 | 187 | 91.63  (83.05-96.07) | 83.24  (73.58-89.85) | 5.47  (3.25-9.20) | 0.10  (0.05-0.22) | 54.37  (16.17-182.76) | 0.94  (0.91-0.95) | 89.66 | 88.91 | 0.40 |
|  |  | Bakula, 2019 | 108 | 67 | 38 | 95 |  |  |  |  |  |  |  |  |  |
|  |  | Gegenava, 2019 | 249 | 16 | 45 | 50 |  |  |  |  |  |  |  |  |  |
|  |  | Teng, 2020 | 183 | 28 | 16 | 147 |  |  |  |  |  |  |  |  |  |
|  |  | Adamichou, 2020 | 630 | 25 | 60 | 376 |  |  |  |  |  |  |  |  |  |
|  |  | Dahlstrom, 2019 | 56 | 14 | 0 | 41 |  |  |  |  |  |  |  |  |  |
|  |  | Lee, 2020 | 330 | 25 | 5 | 312 |  |  |  |  |  |  |  |  |  |
|  | EULAR’19 | Gegenava, 2019 | 257 | 17 | 37 | 49 | 93.64 (88.42-96.60) | 88.77 (77.52-94.77) | 8.34 (3.98-17.46) | 0.07 (0.04-0.13) | 116.39 (39.41-343.67) | 0.97 (0.95-0.98) | 78.12 | 86.74 | 0.11 |
|  |  | Teng, 2020 | 192 | 17 | 7 | 158 |  |  |  |  |  |  |  |  |  |
|  |  | Adamichou, 2020 | 611 | 11 | 79 | 390 |  |  |  |  |  |  |  |  |  |
|  |  | Dahlstrom, 2019 | 52 | 15 | 4 | 40 |  |  |  |  |  |  |  |  |  |
|  |  | Lee, 2020 | 327 | 29 | 8 | 308 |  |  |  |  |  |  |  |  |  |
| ≥ 5% Neurological involvement^#^ | ACR’97 | Sacre, 2021 | 16 | 0 | 1 | 32 | 92.58 (87.15-95.82) | 94.28 (88.59-97.23) | 16.20 (7.70-34.06) | 0.08 (0.04-0.14) | 205.82 (58.52-723.88) | 0.98 (0.96-0.99) | 65.39 | 60.21 | 0.51 |
|  |  | Amezcua-Guerra, 2015 | 97 | 1 | 3 | 99 |  |  |  |  |  |  |  |  |  |
|  |  | Gegenava, 2019 | 261 | 7 | 33 | 59 |  |  |  |  |  |  |  |  |  |
|  |  | Rijnink, 2017 | 110 | 0 | 7 | 32 |  |  |  |  |  |  |  |  |  |
|  |  | Adamichou, 2020 | 591 | 28 | 99 | 273 |  |  |  |  |  |  |  |  |  |
|  |  | Dahlstrom, 2019 | 46 | 10 | 10 | 45 |  |  |  |  |  |  |  |  |  |
|  |  | Lee, 2020 | 320 | 21 | 15 | 316 |  |  |  |  |  |  |  |  |  |
|  | SLICC’12 | Sacre, 2021 | 16 | 0 | 1 | 32 | 96.88 (89.88-99.09) | 92.72 (83.30-97.02) | 13.31 (5.61-31.58) | 0.03 (0.01-0.11) | 395.59  (93.68-1670.45) | 0.99 (0.97-0.99) | 61.03 | 75.01 | 0.72 |
|  |  | Amezcua-Guerra, 2015 | 92 | 1 | 8 | 99 |  |  |  |  |  |  |  |  |  |
|  |  | Gegenava, 2019 | 249 | 16 | 45 | 50 |  |  |  |  |  |  |  |  |  |
|  |  | Rijnink, 2017 | 117 | 3 | 0 | 29 |  |  |  |  |  |  |  |  |  |
|  |  | Adamichou, 2020 | 630 | 25 | 60 | 376 |  |  |  |  |  |  |  |  |  |
|  |  | Dahlstrom, 2019 | 56 | 14 | 0 | 41 |  |  |  |  |  |  |  |  |  |
|  |  | Lee, 2020 | 330 | 25 | 5 | 312 |  |  |  |  |  |  |  |  |  |
|  | EULAR’19 | Sacre, 2021 | 17 | 5 | 0 | 27 | 93.52 (87.20-96.83) | 87.80 (75.27-94.45) | 7.67 (3.60-16.33) | 0.07 (0.04-0.15) | 103.82 (33.46-322.12) | 0.97 (0.95-0.98) | 58.28 | 82.18 | 0.25 |
|  |  | Gegenava, 2019 | 257 | 17 | 37 | 49 |  |  |  |  |  |  |  |  |  |
|  |  | Adamichou, 2020 | 611 | 11 | 79 | 390 |  |  |  |  |  |  |  |  |  |
|  |  | Dahlstrom, 2019 | 52 | 15 | 4 | 40 |  |  |  |  |  |  |  |  |  |
|  |  | Lee, 2020 | 327 | 29 | 8 | 308 |  |  |  |  |  |  |  |  |  |
| ≥50% Anti-dsDNA+ | ACR’97 | Oku,2017 | 206 | 38 | 28 | 215 | 85.10 (72.29-92.59) | 95.33 (91.04-97.62) | 18.23 (9.25-35.91) | 0.16 (0.08-0.30) | 116.60  (40.43-336.31) | 0.97 (0.95-0.98) | 89.24 | 65.65 | 0.45 |
|  |  | Mosca, 2019 | 257 | 19 | 132 | 208 |  |  |  |  |  |  |  |  |  |
|  |  | Sacre, 2021 | 16 | 0 | 1 | 32 |  |  |  |  |  |  |  |  |  |
|  |  | Amezcua-Guerra, 2015 | 97 | 1 | 3 | 99 |  |  |  |  |  |  |  |  |  |
|  |  | Bakula, 2019 | 32 | 3 | 114 | 159 |  |  |  |  |  |  |  |  |  |
|  |  | Assan, 2021 | 38 | 1 | 11 | 48 |  |  |  |  |  |  |  |  |  |
|  |  | Gegenava, 2019 | 261 | 7 | 33 | 59 |  |  |  |  |  |  |  |  |  |
|  |  | Rijnink, 2017 | 110 | 0 | 7 | 32 |  |  |  |  |  |  |  |  |  |
|  |  | Teng, 2020 | 150 | 7 | 49 | 168 |  |  |  |  |  |  |  |  |  |
|  |  | Dahlstrom, 2019 | 46 | 10 | 10 | 45 |  |  |  |  |  |  |  |  |  |
|  |  | Lee, 2020 | 320 | 21 | 15 | 316 |  |  |  |  |  |  |  |  |  |
|  | SLICC’12 | Oku,2017 | 232 | 50 | 2 | 203 | 96.08 (90.34-98.47) | 87.72 (78.93-93.16) | 7.83 (4.40-13.93) | 0.04 (0.02-0.12) | 175.02 (48.37-633.35) | 0.97 (0.95-0.98) | 69.45 | 83.80 | 0.71 |
|  |  | Mosca, 2019 | 325 | 40 | 64 | 187 |  |  |  |  |  |  |  |  |  |
|  |  | Sacre, 2021 | 16 | 0 | 1 | 32 |  |  |  |  |  |  |  |  |  |
|  |  | Amezcua-Guerra, 2015 | 92 | 1 | 8 | 99 |  |  |  |  |  |  |  |  |  |
|  |  | Bakula, 2019 | 108 | 67 | 38 | 95 |  |  |  |  |  |  |  |  |  |
|  |  | Assan, 2021 | 48 | 4 | 1 | 45 |  |  |  |  |  |  |  |  |  |
|  |  | Gegenava, 2019 | 249 | 16 | 45 | 50 |  |  |  |  |  |  |  |  |  |
|  |  | Rijnink, 2017 | 117 | 3 | 0 | 29 |  |  |  |  |  |  |  |  |  |
|  |  | Teng, 2020 | 183 | 28 | 16 | 147 |  |  |  |  |  |  |  |  |  |
|  |  | Dahlstrom, 2019 | 56 | 14 | 0 | 41 |  |  |  |  |  |  |  |  |  |
|  |  | Lee, 2020 | 330 | 25 | 5 | 312 |  |  |  |  |  |  |  |  |  |
|  | EULAR’19 | Sacre, 2021 | 17 | 5 | 0 | 27 | 85.00  (72.00-93.00) | 95.00  (91.00-98.00) | 18.20  (9.30-35.90) | 0.16  (0.08-0.30) | 117.00  (40.00-337.00) | 0.97 (0.95-0.98) | 50.18 | 70.63 | 0.50 |
|  |  | Assan, 2021 | 48 | 2 | 1 | 47 |  |  |  |  |  |  |  |  |  |
|  |  | Gegenava, 2019 | 257 | 17 | 37 | 49 |  |  |  |  |  |  |  |  |  |
|  |  | Teng, 2020 | 192 | 17 | 7 | 158 |  |  |  |  |  |  |  |  |  |
|  |  | Dahlstrom, 2019 | 52 | 15 | 4 | 40 |  |  |  |  |  |  |  |  |  |
|  |  | Lee, 2020 | 327 | 29 | 8 | 308 |  |  |  |  |  |  |  |  |  |
| <20% hemolytic anemia | ACR’97 | Mosca, 2019 | 257 | 19 | 132 | 208 | 84.64 (66.54-93.85) | 95.81 (89.50-98.39) | 20.19 (7.56-53.92) | 0.16 (0.07-0.38) | 125.91 (27.26-581.54) | 0.97 (0.95-0.98) | 89.31 | 64.97 | 0.26 |
|  |  | Sacre, 2021 | 16 | 0 | 1 | 32 |  |  |  |  |  |  |  |  |  |
|  |  | Amezcua-Guerra, 2015 | 97 | 1 | 3 | 99 |  |  |  |  |  |  |  |  |  |
|  |  | Bakula, 2019 | 32 | 3 | 114 | 159 |  |  |  |  |  |  |  |  |  |
|  |  | Gegenava, 2019 | 261 | 7 | 33 | 59 |  |  |  |  |  |  |  |  |  |
|  |  | Rijnink, 2017 | 110 | 0 | 7 | 32 |  |  |  |  |  |  |  |  |  |
|  |  | Adamichou, 2020 | 591 | 28 | 99 | 273 |  |  |  |  |  |  |  |  |  |
|  |  | Dahlstrom, 2019 | 46 | 10 | 10 | 45 |  |  |  |  |  |  |  |  |  |
|  | SLICC’12 | Mosca, 2019 | 325 | 40 | 64 | 187 | 93.47 (83.50-97.59) | 89.58 (76.36-95.81) | 8.97 (3.66-21.98) | 0.07 (0.03-0.20) | 123.07 (25.00-605.86) | 0.97 (0.95-0.98) | 77.55 | 83.54 | 0.58 |
|  |  | Sacre, 2021 | 16 | 0 | 1 | 32 |  |  |  |  |  |  |  |  |  |
|  |  | Amezcua-Guerra, 2015 | 92 | 1 | 8 | 99 |  |  |  |  |  |  |  |  |  |
|  |  | Bakula, 2019 | 108 | 67 | 38 | 95 |  |  |  |  |  |  |  |  |  |
|  |  | Gegenava, 2019 | 249 | 16 | 45 | 50 |  |  |  |  |  |  |  |  |  |
|  |  | Rijnink, 2017 | 117 | 3 | 0 | 29 |  |  |  |  |  |  |  |  |  |
|  |  | Adamichou, 2020 | 630 | 25 | 60 | 376 |  |  |  |  |  |  |  |  |  |
|  |  | Dahlstrom, 2019 | 56 | 14 | 0 | 41 |  |  |  |  |  |  |  |  |  |
|  | EULAR’19 | Sacre, 2021 | 17 | 5 | 0 | 27 | 88.67 (86.11-90.82) | 86.56 (69.00-94.91) | 6.60 (2.63-16.55) | 0.13 (0.10-0.17) | 50.42 (17.07-148.92) | 0.89 (0.86-0.92) | 0.01 | 82.64 | 0.35 |
|  |  | Gegenava, 2019 | 257 | 17 | 37 | 49 |  |  |  |  |  |  |  |  |  |
|  |  | Adamichou, 2020 | 611 | 11 | 79 | 390 |  |  |  |  |  |  |  |  |  |
|  |  | Dahlstrom, 2019 | 52 | 15 | 4 | 40 |  |  |  |  |  |  |  |  |  |

^#^Jia et al. study was omitted because it had a higher rate of neurological involvement than the others, TP; true positive, FP; false positive, FN; false negative, TN; true negative, ACR; American College of Rheumatology, SLICC; Systemic Lupus International Collaborating Clinics, EULAR; European League Against Rheumatism, EU; European Union, ANA; antinuclear antibody, Anti-dsDNA; Anti double-stranded DNA
