# Supplementary material for: Evaluating performance of the 2019 EULAR/ACR, 2012 SLICC, and 1997 ACR criteria for classifying adult-onset and childhood-onset systemic lupus erythematosus: A systematic review and meta-analysis
Source: Front Med (Lausanne). 2022 Dec 22;9:1093213. doi: 10.3389/fmed.2022.1093213 (PMC9813386; doi:10.3389/fmed.2022.1093213)
Supplement: Supplementary file 6 [file Table_6.docx]

**Table S6** Results of subgroup analyses in childhood-onset systemic lupus erythematosus studies.

| **Subgroup** | **Index test** | **Included studies** | **TP** | **FP** | **FN** | **TN** | **Pooled sensitivity (%) (95% CI)** | **Pooled specificity (%) (95% CI)** | **Pooled positive likelihood ratio (95% CI)** | **Pooled negative likelihood ratio (95% CI)** | **Pooled diagnostic ratio (95% CI)** | **The area under the curve (95% CI)** | **Heterogeneity: I^2^** | | **P value of Deeks** |
| --- | --- | --- | --- | --- | --- | --- | --- | --- | --- | --- | --- | --- | --- | --- | --- |
|  |  |  |  |  |  |  |  |  |  |  |  |  | **Sensitivity(%)** | **Specificity (%)** |  |
| Disease duration less than one month | ACR'97 | Arango, 2018 | 43 | 2 | 12 | 53 | 75.56  (66.13-83.04) | 97.01  (89.81-99.17) | 25.31  (6.71-95.49) | 0.25  (0.17-0.36) | 100.48  (20.41-494.80) | 0.90  (0.87-0.93) | 79.30 | 57.96 | 0.22 |
|  |  | Osaku, 2018 | 20 | 1 | 3 | 23 |  |  |  |  |  |  |  |  |  |
|  |  | Alijaberi, 2021 | 81 | 14 | 31 | 91 |  |  |  |  |  |  |  |  |  |
|  |  | Smith, 2021 | 385 | 2 | 97 | 127 |  |  |  |  |  |  |  |  |  |
|  |  | Fonseca, 2019 | 86 | 15 | 36 | 74 |  |  |  |  |  |  |  |  |  |
|  |  | Ma, 2020 | 136 | 0 | 20 | 379 |  |  |  |  |  |  |  |  |  |
|  |  | Abdwani, 2021 | 56 | 2 | 57 | 49 |  |  |  |  |  |  |  |  |  |
|  | SLICC'12 | Arango, 2018 | 49 | 7 | 6 | 48 | 91.76  (84.23-95.87) | 95.84  (87.15-98.74) | 22.07  (6.57-74.11) | 0.09  (0.04-0.17) | 256.84  (43.68-1510.13) | 0.98  (0.96-0.99) | 71.41 | 64.61 | 0.29 |
|  |  | Osaku, 2018 | 23 | 1 | 0 | 23 |  |  |  |  |  |  |  |  |  |
|  |  | Smith, 2021 | 443 | 4 | 39 | 125 |  |  |  |  |  |  |  |  |  |
|  |  | Fonseca, 2019 | 109 | 17 | 13 | 72 |  |  |  |  |  |  |  |  |  |
|  |  | Ma, 2020 | 152 | 1 | 4 | 378 |  |  |  |  |  |  |  |  |  |
|  |  | Abdwani, 2021 | 86 | 3 | 27 | 48 |  |  |  |  |  |  |  |  |  |
|  | EULAR'19 | Alijaberi, 2021 | 95 | 18 | 17 | 87 | 88.26  (80.05-93.37) | 91.77  (78.78-97.10) | 10.73  (3.76-30.61) | 0.13  (0.07-0.23) | 83.84  (18.40-381.97) | 0.95  (0.93-0.96) | 84.38 | 84.92 | 0.16 |
|  |  | Smith, 2021 | 402 | 5 | 80 | 124 |  |  |  |  |  |  |  |  |  |
|  |  | Fonseca, 2019 | 107 | 29 | 15 | 60 |  |  |  |  |  |  |  |  |  |
|  |  | Ma, 2020 | 152 | 6 | 4 | 373 |  |  |  |  |  |  |  |  |  |
|  |  | Abdwani, 2021 | 92 | 4 | 21 | 47 |  |  |  |  |  |  |  |  |  |
| ≥50% ANA+ in the control group | ACR'97 | Fonseca, 2014 | 74 | 8 | 7 | 84 | 72.94  (64.06-80.29) | 93.72  (88.96-96.51) | 11.61  (6.52-20.70) | 0.29  (0.21-0.39) | 40.22  (19.80-81.68) | 0.91  (0.89-0.94) | 86.51 | 68.54 | 0.31 |
|  |  | Alijaberi, 2021 | 81 | 14 | 31 | 91 |  |  |  |  |  |  |  |  |  |
|  |  | Batu, 2021 | 180 | 9 | 82 | 165 |  |  |  |  |  |  |  |  |  |
|  |  | Levinsky, 2021 | 71 | 4 | 41 | 108 |  |  |  |  |  |  |  |  |  |
|  |  | Smith, 2021 | 385 | 2 | 97 | 127 |  |  |  |  |  |  |  |  |  |
|  |  | Fonseca, 2019 | 86 | 15 | 36 | 74 |  |  |  |  |  |  |  |  |  |
|  |  | Abdwani, 2021 | 64 | 2 | 49 | 49 |  |  |  |  |  |  |  |  |  |
|  | SLICC'12 | Fonseca, 2014 | 78 | 11 | 3 | 81 | 91.00  (86.13-94.27) | 91.55  (86.52-94.82) | 10.77  (6.73-17.26) | 0.10  (0.06-0.15) | 109.56  (58.72-204.45) | 0.97  (0.95-0.98) | 73.40 | 64.19 | 0.17 |
|  |  | Batu, 2021 | 250 | 18 | 12 | 156 |  |  |  |  |  |  |  |  |  |
|  |  | Levinsky, 2021 | 92 | 7 | 20 | 106 |  |  |  |  |  |  |  |  |  |
|  |  | Smith, 2021 | 443 | 4 | 39 | 125 |  |  |  |  |  |  |  |  |  |
|  |  | Fonseca, 2019 | 109 | 17 | 13 | 72 |  |  |  |  |  |  |  |  |  |
|  |  | Abdwani, 2021 | 95 | 3 | 18 | 48 |  |  |  |  |  |  |  |  |  |
|  | EULAR'19 | Alijaberi, 2021 | 95 | 18 | 17 | 87 | 86.88  (83.79-89.46) | 89.09  (80.34-94.22) | 7.96  (4.36-14.55) | 0.15  (0.12-0.18) | 54.06  (28.97-100.91) | 0.91  (0.88-0.93) | 40.14 | 81.74 | 0.22 |
|  |  | Batu, 2021 | 240 | 20 | 22 | 154 |  |  |  |  |  |  |  |  |  |
|  |  | Levinsky, 2021 | 93 | 6 | 19 | 107 |  |  |  |  |  |  |  |  |  |
|  |  | Smith, 2021 | 402 | 5 | 80 | 124 |  |  |  |  |  |  |  |  |  |
|  |  | Fonseca, 2019 | 107 | 29 | 15 | 60 |  |  |  |  |  |  |  |  |  |
|  |  | Abdwani, 2021 | 99 | 5 | 14 | 46 |  |  |  |  |  |  |  |  |  |
| ≥35% Renal involvement | ACR'97 | Fonseca, 2014 | 74 | 8 | 7 | 84 | 75.97  (67.21-82.98) | 95.28  (93.34-96.68) | 16.10  (11.62-22.30) | 0.25  (0.18-0.35) | 63.84  (40.44-100.78) | 0.96  (0.94-0.98) | 79.92 | 10.31 | 0.92 |
|  |  | Sag, 2014 | 118 | 8 | 36 | 115 |  |  |  |  |  |  |  |  |  |
|  |  | Arango, 2018 | 43 | 2 | 12 | 53 |  |  |  |  |  |  |  |  |  |
|  |  | Osaku, 2018 | 20 | 1 | 3 | 23 |  |  |  |  |  |  |  |  |  |
|  |  | Batu, 2021 | 180 | 9 | 82 | 165 |  |  |  |  |  |  |  |  |  |
|  |  | Levinsky, 2021 | 71 | 4 | 41 | 108 |  |  |  |  |  |  |  |  |  |
|  |  | Smith, 2021 | 385 | 2 | 97 | 127 |  |  |  |  |  |  |  |  |  |
|  |  | Abdwani, 2021 | 64 | 2 | 49 | 49 |  |  |  |  |  |  |  |  |  |
|  | SLICC'12 | Fonseca, 2014 | 78 | 11 | 3 | 81 | 93.66  (88.66-96.54) | 91.55  (88.30-93.97) | 11.09  (8.14-15.11) | 0.07  (0.04-0.12) | 160.08  (96.28-266.16) | 0.96  (0.94-0.98) | 68.16 | 36.12 | 0.57 |
|  |  | Sag, 2014 | 152 | 18 | 2 | 105 |  |  |  |  |  |  |  |  |  |
|  |  | Arango, 2018 | 49 | 7 | 6 | 48 |  |  |  |  |  |  |  |  |  |
|  |  | Osaku, 2018 | 23 | 1 | 0 | 23 |  |  |  |  |  |  |  |  |  |
|  |  | Batu, 2021 | 250 | 18 | 12 | 156 |  |  |  |  |  |  |  |  |  |
|  |  | Levinsky, 2021 | 92 | 7 | 20 | 106 |  |  |  |  |  |  |  |  |  |
|  |  | Smith, 2021 | 443 | 4 | 39 | 125 |  |  |  |  |  |  |  |  |  |
|  |  | Abdwani, 2021 | 95 | 3 | 18 | 48 |  |  |  |  |  |  |  |  |  |
|  | EULAR'19 | Batu, 2021 | 240 | 20 | 22 | 154 | 86.93  (82.51-90.36) | 93.04  (88.61-95.83) | 12.49  (7.73-20.16) | 0.14  (0.11-0.19) | 88.87  (56.90-138.81) | 0.95  (0.93-0.97) | 58.84 | 46.92 | 0.36 |
|  |  | Levinsky, 2021 | 93 | 6 | 19 | 107 |  |  |  |  |  |  |  |  |  |
|  |  | Smith, 2021 | 402 | 5 | 80 | 124 |  |  |  |  |  |  |  |  |  |
|  |  | Abdwani, 2021 | 99 | 5 | 14 | 46 |  |  |  |  |  |  |  |  |  |
| ≥ 5% Neurological involvement | ACR'97 | Fonseca, 2014 | 74 | 8 | 7 | 84 | 76.44  (68.65-82.77) | 95.72  (89.16-98.38) | 17.86  (6.59-48.42) | 0.25  (0.18-0.34) | 72.56  (21.69-242.75) | 0.90  (0.87-0.92) | 86.43 | 76.86 | 0.13 |
|  |  | Sag, 2014 | 118 | 8 | 36 | 115 |  |  |  |  |  |  |  |  |  |
|  |  | Aljaberi, 2021 | 81 | 14 | 31 | 91 |  |  |  |  |  |  |  |  |  |
|  |  | Batu, 2021 | 180 | 9 | 82 | 165 |  |  |  |  |  |  |  |  |  |
|  |  | Smith, 2021 | 385 | 2 | 97 | 127 |  |  |  |  |  |  |  |  |  |
|  |  | Fonseca, 2019 | 86 | 15 | 36 | 74 |  |  |  |  |  |  |  |  |  |
|  |  | Ma, 2020 | 136 | 0 | 20 | 379 |  |  |  |  |  |  |  |  |  |
|  |  | Abdwani, 2021 | 64 | 2 | 49 | 49 |  |  |  |  |  |  |  |  |  |
|  | SLICC'12 | Fonseca, 2014 | 78 | 11 | 3 | 81 | 94.53  (90.32-96.97) | 93.79  (85.21-97.54) | 15.23  (6.13-37.80) | 0.06  (0.03-0.11) | 260.98  (78.05-872.60) | 0.98  (0.96-0.99) | 75.62 | 82.29 | 0.19 |
|  |  | Sag, 2014 | 152 | 18 | 2 | 105 |  |  |  |  |  |  |  |  |  |
|  |  | Batu, 2021 | 250 | 18 | 12 | 156 |  |  |  |  |  |  |  |  |  |
|  |  | Smith, 2021 | 443 | 4 | 39 | 125 |  |  |  |  |  |  |  |  |  |
|  |  | Fonseca, 2019 | 109 | 17 | 13 | 72 |  |  |  |  |  |  |  |  |  |
|  |  | Ma, 2020 | 152 | 1 | 4 | 378 |  |  |  |  |  |  |  |  |  |
|  |  | Abdwani, 2021 | 95 | 3 | 18 | 48 |  |  |  |  |  |  |  |  |  |
|  | EULAR'19 | Aljaberi, 2021 | 95 | 18 | 17 | 87 | 89.61  (83.95-93.44) | 90.94  (80.27-96.12) | 9.89  (4.23-23.09) | 0.11  (0.07-0.19) | 86.57  (25.22-297.21) | 0.95  (0.93-0.97) | 79.94 | 85.91 | 0.20 |
|  |  | Batu, 2021 | 240 | 20 | 22 | 154 |  |  |  |  |  |  |  |  |  |
|  |  | Smith, 2021 | 402 | 5 | 80 | 124 |  |  |  |  |  |  |  |  |  |
|  |  | Fonseca, 2019 | 107 | 29 | 15 | 60 |  |  |  |  |  |  |  |  |  |
|  |  | Ma, 2020 | 152 | 6 | 4 | 373 |  |  |  |  |  |  |  |  |  |
|  |  | Abdwani, 2021 | 99 | 5 | 14 | 46 |  |  |  |  |  |  |  |  |  |
| ≥50% Anti-dsDNA+ | ACR'97 | Fonseca, 2014 | 74 | 8 | 7 | 84 | 78.97  (70.89-85.27) | 97.16  (93.52-98.78) | 27.84  (11.75-65.94) | 0.22  (0.15-0.31) | 128.60  (45.38-364.49) | 0.94  (0.92-0.96) | 78.49 | 55.05 | 0.44 |
|  |  | Sag, 2014 | 118 | 8 | 36 | 115 |  |  |  |  |  |  |  |  |  |
|  |  | Arango, 2018 | 43 | 2 | 12 | 53 |  |  |  |  |  |  |  |  |  |
|  |  | Osaku, 2018 | 20 | 1 | 3 | 23 |  |  |  |  |  |  |  |  |  |
|  |  | Batu, 2021 | 180 | 9 | 82 | 165 |  |  |  |  |  |  |  |  |  |
|  |  | Smith, 2021 | 385 | 2 | 97 | 127 |  |  |  |  |  |  |  |  |  |
|  |  | Ma, 2020 | 136 | 0 | 20 | 379 |  |  |  |  |  |  |  |  |  |
|  |  | Abdwani, 2021 | 64 | 2 | 49 | 49 |  |  |  |  |  |  |  |  |  |
|  | SLICC'12 | Fonseca, 2014 | 78 | 11 | 3 | 81 | 95.08  (91.07-97.35) | 94.45  (88.04-97.52) | 17.14  (7.74-37.94) | 0.05  (0.03-0.10) | 329.24  (111.34-973.54) | 0.98  (0.97-0.99) | 61.63 | 71.44 | 0.33 |
|  |  | Sag, 2014 | 152 | 18 | 2 | 105 |  |  |  |  |  |  |  |  |  |
|  |  | Arango, 2018 | 49 | 7 | 6 | 48 |  |  |  |  |  |  |  |  |  |
|  |  | Osaku, 2018 | 23 | 1 | 0 | 23 |  |  |  |  |  |  |  |  |  |
|  |  | Batu, 2021 | 250 | 18 | 12 | 156 |  |  |  |  |  |  |  |  |  |
|  |  | Smith, 2021 | 443 | 4 | 39 | 125 |  |  |  |  |  |  |  |  |  |
|  |  | Ma, 2020 | 152 | 1 | 4 | 378 |  |  |  |  |  |  |  |  |  |
|  |  | Abdwani, 2021 | 95 | 3 | 18 | 48 |  |  |  |  |  |  |  |  |  |
|  | EULAR'19 | Batu, 2021 | 240 | 20 | 22 | 154 | 91.26  (83.30-95.62) | 94.81  (88.52-97.74) | 17.59  (7.51-41.23) | 0.09  (0.05-0.19) | 190.78  (47.93-759.40) | 0.98  (0.96-0.99) | 85.39 | 71.91 | 0.63 |
|  |  | Smith, 2021 | 402 | 5 | 80 | 124 |  |  |  |  |  |  |  |  |  |
|  |  | Ma, 2020 | 152 | 6 | 4 | 373 |  |  |  |  |  |  |  |  |  |
|  |  | Abdwani, 2021 | 99 | 5 | 14 | 46 |  |  |  |  |  |  |  |  |  |
| ≥50% Hematological involvement  or  ≥20% hemolytic anemia | ACR'97 | Fonseca, 2014 | 74 | 8 | 7 | 84 | 76.07  (67.84-82.73) | 96.15  (90.78-98.45) | 19.77  (7.80-50.13) | 0.25  (0.18-0.34) | 79.44  (25.70-245.59) | 0.91  (0.88-0.93) | 82.34 | 67.88 | 0.28 |
|  |  | Osaku, 2018 | 20 | 1 | 3 | 23 |  |  |  |  |  |  |  |  |  |
|  |  | Aljaberi, 2021 | 81 | 14 | 31 | 91 |  |  |  |  |  |  |  |  |  |
|  |  | Batu, 2021 | 180 | 9 | 82 | 165 |  |  |  |  |  |  |  |  |  |
|  |  | Levinsky, 2021 | 71 | 4 | 41 | 108 |  |  |  |  |  |  |  |  |  |
|  |  | Smith, 2021 | 385 | 2 | 97 | 127 |  |  |  |  |  |  |  |  |  |
|  |  | Fonseca, 2019 | 86 | 15 | 36 | 74 |  |  |  |  |  |  |  |  |  |
|  |  | Ma, 2020 | 136 | 0 | 20 | 379 |  |  |  |  |  |  |  |  |  |
|  |  | Abdwani, 2021 | 64 | 2 | 49 | 49 |  |  |  |  |  |  |  |  |  |
|  | SLICC'12 | Fonseca, 2014 | 78 | 11 | 3 | 81 | 92.94  (88.15-95.89) | 94.82  (88.50-97.75) | 17.94  (7.77-41.43) | 0.07  (0.04-0.13) | 241.03  (72.94-796.43) | 0.98  (0.96-0.99) | 68.62 | 71.82 | 0.36 |
|  |  | Osaku, 2018 | 23 | 1 | 0 | 23 |  |  |  |  |  |  |  |  |  |
|  |  | Batu, 2021 | 250 | 18 | 12 | 156 |  |  |  |  |  |  |  |  |  |
|  |  | Levinsky, 2021 | 92 | 7 | 20 | 106 |  |  |  |  |  |  |  |  |  |
|  |  | Smith, 2021 | 443 | 4 | 39 | 125 |  |  |  |  |  |  |  |  |  |
|  |  | Fonseca, 2019 | 109 | 17 | 13 | 72 |  |  |  |  |  |  |  |  |  |
|  |  | Ma, 2020 | 152 | 1 | 4 | 378 |  |  |  |  |  |  |  |  |  |
|  |  | Abdwani, 2021 | 95 | 3 | 18 | 48 |  |  |  |  |  |  |  |  |  |
|  | EULAR'19 | Aljaberi, 2021 | 95 | 18 | 17 | 87 | 88.84  (83.73-92.48) | 91.62  (83.11-96.04) | 10.60  (5.00-22.48) | 0.12  (0.08-0.19) | 86.96  (30.40-248.79) | 0.95  (0.93-0.96) | 78.02 | 84.35 | 0.15 |
|  |  | Batu, 2021 | 240 | 20 | 22 | 154 |  |  |  |  |  |  |  |  |  |
|  |  | Levinsky, 2021 | 93 | 6 | 19 | 107 |  |  |  |  |  |  |  |  |  |
|  |  | Smith, 2021 | 402 | 5 | 80 | 124 |  |  |  |  |  |  |  |  |  |
|  |  | Fonseca, 2019 | 107 | 29 | 15 | 60 |  |  |  |  |  |  |  |  |  |
|  |  | Ma, 2020 | 152 | 6 | 4 | 373 |  |  |  |  |  |  |  |  |  |
|  |  | Abdwani, 2021 | 99 | 5 | 14 | 46 |  |  |  |  |  |  |  |  |  |

TP; true positive, FP; false positive, FN; false negative, TN; true negative, ACR; American College of Rheumatology, SLICC; Systemic Lupus International Collaborating Clinics, EULAR; European League Against Rheumatism
