# Supplementary figures and images for: Evaluating performance of the 2019 EULAR/ACR, 2012 SLICC, and 1997 ACR criteria for classifying adult-onset and childhood-onset systemic lupus erythematosus: A systematic review and meta-analysis
Source: Front Med (Lausanne). 2022 Dec 22;9:1093213. doi: 10.3389/fmed.2022.1093213 (PMC9813386; doi:10.3389/fmed.2022.1093213)

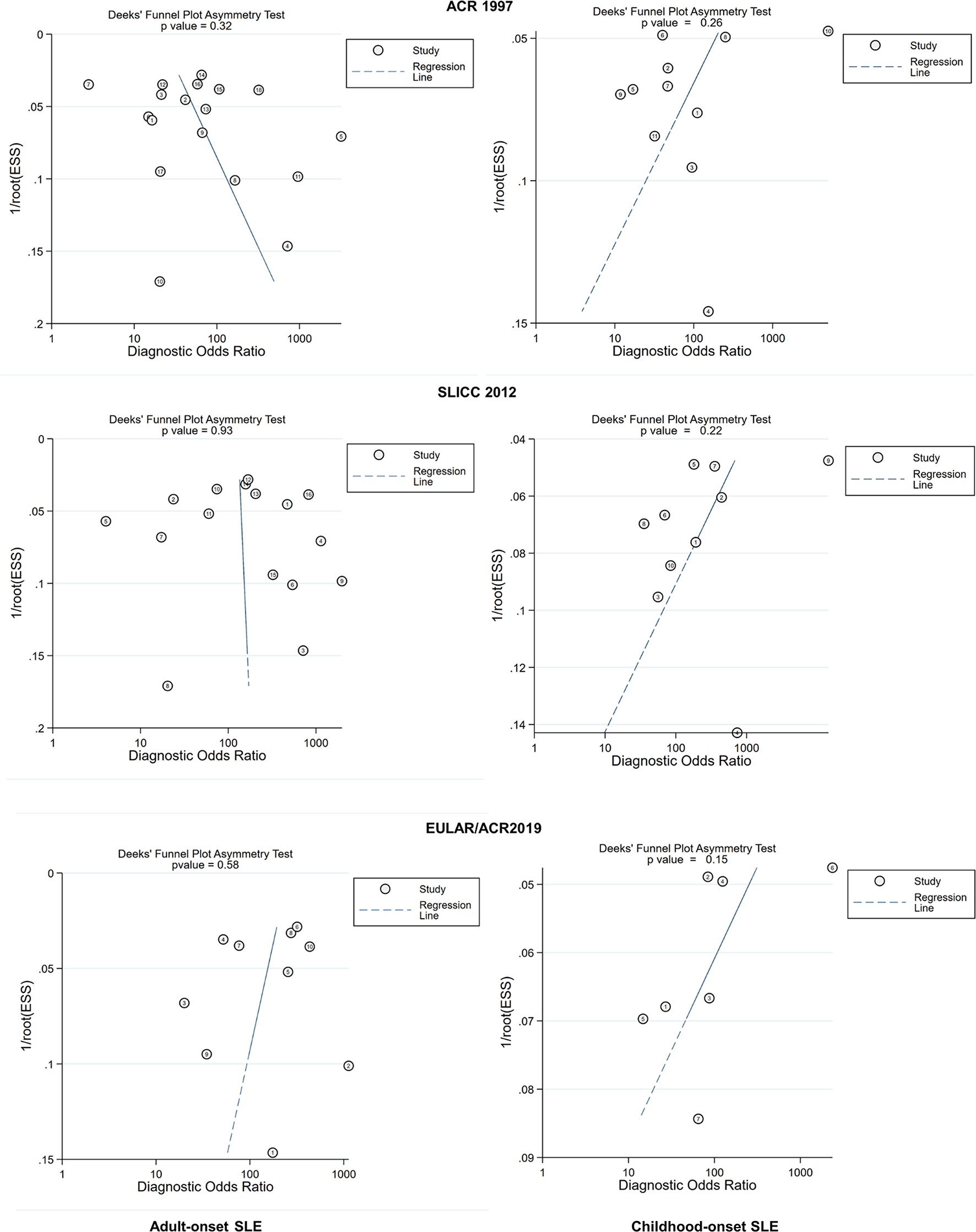

Supplement: Supplementary Figure 1 — Deeks’ funnel plots to evaluate the publication bias of all included adult-onset and childhood-onset systemic lupus erythematosus studies. [file Image_1.TIF]
